# Comparison of Machine Learning Techniques for Mortality Prediction in a Prospective Cohort of Older Adults

**DOI:** 10.3390/ijerph182312806

**Published:** 2021-12-04

**Authors:** Salvatore Tedesco, Martina Andrulli, Markus Åkerlund Larsson, Daniel Kelly, Antti Alamäki, Suzanne Timmons, John Barton, Joan Condell, Brendan O’Flynn, Anna Nordström

**Affiliations:** 1 Tyndall National Institute, University College Cork, Lee Maltings Complex, Dyke Parade, T12R5CP Cork, Ireland; andrullimartina@gmail.com (M.A.); john.barton@tyndall.ie (J.B.); brendan.oflynn@tyndall.ie (B.O.); 2 Department of Public Health and Clinical Medicine, Section of Sustainable Health, Umeå University, SE-901 87 Umeå, Sweden; Markus.Akerlund.Larsson@regionvasterbotten.se (M.Å.L.); anna.h.nordstrom@umu.se (A.N.); 3 School of Computing, Engineering and Intelligent Systems, Ulster University, Londonderry BT48 7JL, UK; d.kelly@ulster.ac.uk (D.K.); j.condell@ulster.ac.uk (J.C.); 4 Department of Physiotherapy, Karelia University of Applied Sciences, Tikkarinne 9, FI-80200 Joensuu, Finland; Antti.Alamaki@karelia.fi; 5 Centre for Gerontology and Rehabilitation, University College Cork, T12XH60 Cork, Ireland; S.Timmons@ucc.ie; 6 School of Sport Sciences, UiT the Arctic University of Norway, 9037 Tromsø, Norway

**Keywords:** ageing, all-cause mortality, imbalanced data, machine learning, mortality prediction, older adults, prediction models

## Abstract

As global demographics change, ageing is a global phenomenon which is increasingly of interest in our modern and rapidly changing society. Thus, the application of proper prognostic indices in clinical decisions regarding mortality prediction has assumed a significant importance for personalized risk management (i.e., identifying patients who are at high or low risk of death) and to help ensure effective healthcare services to patients. Consequently, prognostic modelling expressed as all-cause mortality prediction is an important step for effective patient management. Machine learning has the potential to transform prognostic modelling. In this paper, results on the development of machine learning models for all-cause mortality prediction in a cohort of healthy older adults are reported. The models are based on features covering anthropometric variables, physical and lab examinations, questionnaires, and lifestyles, as well as wearable data collected in free-living settings, obtained for the “Healthy Ageing Initiative” study conducted on 2291 recruited participants. Several machine learning techniques including feature engineering, feature selection, data augmentation and resampling were investigated for this purpose. A detailed empirical comparison of the impact of the different techniques is presented and discussed. The achieved performances were also compared with a standard epidemiological model. This investigation showed that, for the dataset under consideration, the best results were achieved with Random UnderSampling in conjunction with Random Forest (either with or without probability calibration). However, while including probability calibration slightly reduced the average performance, it increased the model robustness, as indicated by the lower 95% confidence intervals. The analysis showed that machine learning models could provide comparable results to standard epidemiological models while being completely data-driven and disease-agnostic, thus demonstrating the opportunity for building machine learning models on health records data for research and clinical practice. However, further testing is required to significantly improve the model performance and its robustness.

## 1. Introduction

According to a UK study carried out in 2010, 15% of the European population (those aged >65 years) consumed approximately 60% of healthcare resources, and the estimated healthcare expenditure associated with long-term care is expected to rise by 315% by 2051 if medical approaches on the older adults remain unchanged [1]. Europe is the world’s oldest continent in demographic terms and, by 2060, 155 million Europeans, representing 30% of the total population, will be aged 65 or older [2].

As a result of the global phenomenon that is rapid ageing, researchers are aiming at extending the duration of the human lifecycle while, at the same time, minimizing the overall healthcare cost associated with this longevity. An example of this interest is the development of a prediction of the person’s risk of death [3,4,5]. The process of progressive deterioration of various physiological systems in the human body increases while aging and, as a result, the risk of disability, mortality, and morbidity increases steadily over the adult lifetime [6].

However, the aging process cannot be considered as standardized and, for this reason, the diagnosis of a disease or any prediction regarding mortality significantly varies across individuals, even when considering people with the same age [7]. This is particularly evident in the older adult population, where there is a considerable diversity in health status, functional limitations, and medication interventions [7].

The application of proper prognostic indices in clinical decisions regarding mortality prediction has assumed a significant importance for personalized risk management (i.e., identifying patients who are at high or low risk of death) and to ensure effective healthcare services to patients [8].

Several clinical indices or scores have been proposed in literature to predict mortality. Some examples are the Acute Physiology and Chronic Health Evaluation (APACHE) [9], the Simplified Acute Physiology Score (SAPS) [10], the Mortality Probability Model (MPM) [11], the European System for Cardiac Operative Risk Evaluation (EuroSCORE) [12], and many more. However, those scores are usually defined and are applied to specific cohorts, e.g., subjects in the Intensive Care Unit (ICU), who are hospitalized or in nursing homes, who are post-surgery, following a traumatic event (i.e., myocardial infarction, subarachnoid hemorrhage, major blunt trauma, etc.), or with specific conditions (e.g., diabetes, sepsis). Several geriatric scores, originally defined for functional analysis in older adults, have also been correlated to mortality prediction, such as the Katz Index of Independence in Activities of Daily Living [13], the Lawton Instrumental Activities of Daily Living Scale [14], the Barthel Index [15,16], the Clinical Frailty Score [17], and so on.

Even though all these scores are well-established, easy to use and to understand, these models were defined decades ago and do not consider the changes which have taken place in recent years in terms of patient care characteristics (i.e., mean age, number and severity of chronic diseases presented), healthcare delivery models and outcomes that occurred in the meantime [18].

Moreover, demographic and actuarial literature has investigated mortality prediction modelling and forecasting [19,20,21] via an increasing use of statistical methods generally divided into three main approaches: expectation, extrapolation, and explanation. However, standard mortality models in the actuarial literature may not be able to identify patterns which instead could be captured via artificial intelligence methods [22].

In this context, machine learning (ML) has the potential to transform several aspects of patient care; these include the ability to guarantee affordable and less invasive surgery options, provide holistic therapies, and develop new care models. The adoption of ML in healthcare has seen a rapid growth in diagnostics and prognosis [23,24,25,26]. ML mortality risk modelling has been generally applied on patients with existing pathologies (e.g., cancer [27], acute coronary syndrome [28], kidney disease [29,30], pulmonary disease [31]), in ICU or critical care [32,33] or following post-operative surgeries (such as stem-cell transplantation [34]). However, only very few studies [35,36,37] have investigated ML modelling for all-cause mortality prediction in a general population cohort without directly studying the impact that different techniques for handling imbalanced datasets had on the overall performance.

This work investigates the development of an ML model able to predict mortality in a two to seven year time frame in a cohort of healthy older adults, and aims to provide a comprehensive comparison on the impact shown by the standard techniques for imbalanced datasets on the overall model. For this purpose, several ML techniques including feature engineering, feature selection, data augmentation, resampling, and so on were investigated. The predictive model is based on features covering anthropometric variables, physical and lab examinations, questionnaires, and lifestyles, as well as wearable data collected in free-living settings. Finally, a comparison of the achieved performance with a standard epidemiological model was also undertaken.

## 2. Methods

### 2.1. Dataset Description

The dataset used in this investigation was provided by the “Healthy Ageing Initiative” (HAI) study [38], conducted in Umeå, Sweden. HAI is an ongoing primary prevention study conducted at a single clinic with the aim of identifying traditional and potentially new risk factors for cardiovascular disorders, falls, and fractures among 70-year-olds in Umeå [39]. The eligibility criteria were: residence in Umeå municipality and an age of exactly 70. There were no exclusion criteria, and population registers were used for recruitment.

For this work, the data collected in the period from January 2013 to December 2017 were considered. The data collection involved a 3-h health examination for each participant, who was then asked to wear an ActiGraph GT3X+ (ActiGraph, LLC, Pensacola, FL, USA) [40] on the hip for one week. The status of the subjects was monitored using population registers in order to know which patients passed away in the time between their data collection and the end of the study date (31 December 2019).

The study aimed to generate a dataset including various and heterogeneous features to evaluate all the possible aspects that influenced older people’s daily life, which are summarized below:

- Anthropometry: gender, height, weight, hip and waist circumference, body mass index (BMI);
- Medication/Medical history: diabetes, rheumatoid arthritis, secondary osteoporosis, stroke, heart infarction, blood pressure medication, statins, glucocorticoids, previous fracture, previous fall, parent fractured hip;
- Lab analysis: systolic-diastolic blood pressure, plasma glucose, total-HDL-LDL cholesterol, triglycerides, heart rate, manual muscle test (MMT), peak expiratory flow, hand grip strength non-dominant hand, time up and go (TUG);
- Questionnaires/Lifestyles: Mental health/depression (Geriatric Depression Scale-GDS [41]), tobacco and alcohol consumption, AUDIT-C score [42], physical activity and exercise (IPAQ–International Physical Activity Questionnaire [43]);
- Lab tools: GAITRite [44] gait analysis data (i.e., step time, step length, etc.), balance test (sway with full and no vision), bone scans of the non-dominant cortical, trabecular, radius and tibia (via computed tomography-pQCT), total T-score, and analysis of fat and lean mass of various body parts (via X-ray absorptiometry-DXA);
- ActiGraph 1-week accelerometer data (e.g., steps taken, time in light, sedentary, moderate, vigorous activities, energy expenditure, etc.). For the data to be acceptable the minimum wear time per day was 600 min, for at least four days.

In contrast with standard datasets on mortality prediction, this dataset also includes data collected from wearable sensors based on the suggestion by Burnham et al. in [45] that data obtained from wearable technology could be predictive of, or significantly associated with, health outcomes.

The overall dataset consisted of 156 parameters for 2291 recruited participants. Of those participants, 92 subjects (approx. 4%) died in the two to seven years follow-up period. For this reason, several imbalanced techniques have been considered in the implementation of the ML modelling.

### 2.2. Machine Learning Modelling

The following section describes a number of approaches considered during the implementation of the ML modelling for this highly imbalanced dataset [46]. The experiments were implemented in Python 3 (Python Software Foundation, Wilmington, DE, USA). The dataset was split into three partitions: training, validation, and test sets. The test was obtained from the 30% of the whole available data, while the remaining 70% was split again into 30% assigned to the validation set and 70% to the training set. The division was stratified to guarantee that the proportion between positive (“subjects who passed away by the end of the study”) and negative (“subjects who were still alive”) cases was the same in every set.

#### 2.2.1. Data Pre-Processing

Categorical features (e.g., “IPAQ” results) were transformed utilizing one-hot encoding. Those features characterized by “True/False” values were transformed according to a binary association (True was set to 1, False to 0). Age was discarded from the analysis as all the recruited subjects were 70 years old. Moreover, since the variables presented different scales, a normalization process was performed by estimating the mean and the standard deviation of each feature and with the normalized variable obtained by subtracting the feature mean and dividing it by the correspondent standard deviation. The means and standard deviations were calculated for the training set and then used on the validation and test sets to avoid any possible leakage.

Finally, in case of missing entries in the dataset (e.g., because a subject did not complete a specific test), imputation was performed using the mean of the feature for continuous variables and the mode for categorical ones. Again, the calculated means/modes were obtained for the training set and then used on the validation and test sets to avoid any possible leakage. Only 0.124% of the data was missing overall, which is a negligible value. The features with the most missing values were LDL cholesterol (data missing from 0.35% of subjects), peak expiratory flow (data missing from 0.26% of subjects), and AUDIT-C score (data missing from 11.78% of subjects).

#### 2.2.2. Feature Engineering

Feature engineering is the process of using domain knowledge to create new variables to increase the predictive power and accuracy of the model. Given the advanced age of the participants, a first analysis on the implementation of a frailty index was conducted from the existing variables. Frailty is the clinically recognized state of increased vulnerability, related to the ageing process, which is manifested through a decline in the body’s physical and psychological reserves. In order to quantify frailty state, it is required to construct a frailty index (FI), based on the accumulation of a variety of functional deficits. Many frailty indices are available in the literature [47]; however, this work adopted the FI implemented by Williams et al. [48] on the UK Biobank dataset, showing the use of multiple features to quantify the FI. The FI is obtained by a combination of variables related to deficits, where for each condition a value is assigned (0—absence, 1—severe presence). The final FI value of each participant is computed as the sum of deficits accrued, divided by the total number of deficits considered. While the original study considered 49 traits covering health, the presence of diseases and disabilities, and mental well-being, in this paper the index was created based on only the 10 common traits which were also included in the available dataset (e.g., MMT score, GDS, diabetes, heart infarction, high blood pressure, high cholesterol, total T-score, secondary osteoporosis, rheumatoid arthritis, and previous fractures).

A Mortality Index (MI) was also developed based on the work of Kim et al. [49]. MI can be seen as a lifestyle index generated to detect the effects on health of different behavioral factors. Each feature used for developing the index represented a risk factor with a certain point score, depending on the value assumed by the feature itself or by the characteristics of the participants. The MI for each participant was evaluated by adding up these points, obtaining a total risk score ranging from 0 to 21 points. While the study described in [49] was based on nine risk factors (e.g., age, male sex, smoking, diabetes, systolic blood pressure, triglyceride, total cholesterol, white blood cell count, and hemoglobin), in this work the MI was developed considering only the first seven factors, as the last two were not included in the dataset under consideration in this analysis.

#### 2.2.3. Models and Hyper-Parameters Tuning

The following models with the related hyper-parameters were considered for the analysis:

- Logistic Regression (LR): penalty = [‘l1’, ‘l2’], C = [1e-3, 1e-2.33, 1e-1.66, 1e-1, 1e-0.33, 1e0.33, 1e1, 1e1.66, 1e2.33, 1e3];
- Decision Tree (DT): criterion = [‘entropy’, ‘gini’], max_depth = [1–10], min_samples_leaf = [1–5], min_samples_split = [2–20];
- Random Forest (RF): N_estimators = [10–100], max_features = [‘auto’, ‘sqrt’], min_sample_leaf = [1–4];
- Adaptive Boosting (AdaBoost): N_estimators = [10–100], learning_rate = [1e-4, 1e-3, 1e-2, 1e-1, 2e-1, 3e-1], base estimator = decision tree.

A grid search was employed on the training set to attain optimal values for the hyper-parameters. For each combination of hyper-parameters’ values, a fivefold stratified cross-validation (CV) procedure was carried out. The combination of hyper-parameters that returned estimates with the higher score was considered to be the optimum. The models were evaluated on the validation set to show that they were able to generalize their predictions with the selected set of hyper-parameters’ values without over-fitting. Consecutively, training and validation sets were merged into a single new training set, and the models with the selected hyper-parameters’ values were re-fit on this new training set and finally evaluated on the test set. The prediction scores for both training and test sets were calculated.

The scoring metric utilized to optimize the overall model performance is the AUC-ROC; however, given the highly imbalanced dataset available, other useful metrics (AUC-PR, Brier score, F1 score, accuracy, precision, and recall) are also provided for evaluation.

#### 2.2.4. Feature Selection and Outlier Removal

Feature selection is essential to reduce the risk of over-fitting, especially when using a dataset with very high dimensionality. Among all the well-known feature selection methods [50], the algorithm chosen in this work was the Forward Selection Component Analysis (FSCA) [51]. FSCA is a technique performing variable selection and dimensionality reduction at the same time. As shown in [52], FSCA can also be successfully adopted to build interpretable and robust systems for anomaly detection. This is possible because FSCA works differently from other feature selection techniques since it focuses on selecting those features that can discriminate more easily between the two different classes. The pseudo-code for FSCA is shown in Figure S1A (Supplementary Data 1). The main limitation of the method is represented by the need to define the value of features to select K *a priori*. For this analysis, values of K between five and 20 were taken into account.

Outlier removal is also an important step required to minimize over-fitting. In this work, Isolation Forest was used to detect and remove possible participant outliers in the training set. Isolation Forest is a simple but effective method for identifying possible anomalies in the dataset requiring few parameters [53]. In particular, the contamination level was set to 0.1, while a total of 50 decision trees have been used.

#### 2.2.5. Monte Carlo Data Augmentation

In case of highly imbalanced datasets, it could be helpful to generate new synthetic samples. This can be achieved by means of data augmentation, a process that increases the amount of training data using information available from the training data itself. While data augmentation is well-known in image-related problems, its application to tabular data or electronic health records (EHRs) is less obvious. This is because it is challenging to create records for new synthetic patients which could be recognized as data describing real patients [54].

In this work, the data augmentation algorithm chosen was the Monte Carlo approach proposed in [55]. This approach has been investigated for its ease-of-use and optimal results achieved in other contexts. In particular, the algorithm computes an initial matrix indicated as P, having as many rows as the desired new synthetic samples, and where each feature value is randomly obtained within a range defined by the minimum and maximum of the original feature itself. To preserve the fundamental characteristics of the original dataset, the algorithm then uses geometric distances and K-Nearest Neighbors to compute new samples labels. Once each row of the matrix is properly labelled, the algorithm returns P as final matrix containing all the new synthetic samples. Finally, the generated variables have been properly treated to increase the realistic simulation of the synthetic data (e.g., by forcing the categorical variables to remain categorical). Figure S2A (Supplementary Data 1) shows the pseudo-code for the process.

#### 2.2.6. Over/Under-Sampling, Cost-Sensitive Learning, and Probability Calibration

A technique generally used to deal with highly imbalanced datasets is resampling. When resampling is applied, the data used for model training change by under-sampling the majority class, over-sampling the minority class, or both. In this work, several techniques have been considered covering over-sampling (e.g., SMOTE [56], ADASYN [57]), under-sampling (e.g., RUS [58]), and over/under-sampling (e.g., SMOTEENN [59]). SMOTE, ADASYN, RUS, and SMOTEENN were chosen as those techniques are the most widespread sampling techniques in literature.

While generally standard ML classifiers consider equally all the misclassification errors computed by the model, in an imbalanced classification it is common to rely on cost-sensitive learning approaches to consider all the misclassification costs while training the ML model [60]. A simple approach to include cost-sensitive learning in the base classifiers is by introducing a “class_weight” hyper-parameter which controls the ratio of class weight between samples of the majority class and samples of the minority class. A higher ratio gives more emphasis to the minority class. In this work the ratios considered were 1:10, 1:20, and 1:100. The combination of sampling technique and cost-sensitive learning is usually performed to handle the data imbalance while achieving high recall and reasonable precision [61].

Finally, another important aspect to be considered is the ability of ML models to predict a probability or probability-like score for class samples. It is usually desired that the distribution of the predicted probability is similar to the distribution of the observed one in the training data. If this condition is not feasible, the model may be over-confident in some cases and under-confident in other cases, especially in cases of highly imbalanced datasets. Hence, probability calibration is required and sometimes needs to be forced by rescaling the achieved probability values to match the distribution observed in the training data [62]. Calibrating probability may be even more essential if resampling techniques are used, as sampling can introduce a bias in the posterior probability [63]. In this work, isotonic regression was used to achieve this purpose via a 3-fold CV.

## 3. Results

All the techniques previously discussed are analyzed in this section. All the possible combinations across the techniques and models have been investigated to ensure a thorough performance evaluation. All the tested models have in common the data pre-processing steps, feature engineering, FSCA, and Isolation Forest, to prevent over-fitting. The number of chosen features K selected by FSCA has been changed properly, together with the models’ hyper-parameters, again to prevent over-fitting while achieving the optimal performance. Furthermore, the analysis has been repeated six times with different data split across training, validation, and test sets to show the repeatability of the model performance. The results in this section are reported as the mean performance of every model for every metric considered as well as its 95% confidence interval (C.I.). It is important to underline that special attention was paid to make sure that none of the developed model was affected by overfitting, as it is evident from the limited difference in performance between training and testing results.

### 3.1. Epidemiological Model

A standard epidemiological model based on a multivariate Cox proportional-hazards model was developed to provide a benchmark for the evaluation of the ML models implemented. The model was developed based on the methodology reported in [36]. Firstly, all the variables that in the training set reported a *p*-value larger than 0.1 when compared against the label were removed. Moreover, to eliminate any multi-collinearity in the training dataset, a stepwise approach was adopted to remove features with a Variance Inflation Factor (VIF) larger than 5. This process preserved only 28 variables from the dataset. Finally, a systematic backwards elimination method was performed based on the *p*-value of each feature, removing at every step the feature with the largest *p*-value. At each iteration, the AUC-ROC was checked, and the process was repeated until a significant loss in performance occurred. The optimal model was identified as the model with the least number of variables without a significant decrease in model performance. Moreover, the model was also validated on the test set to ensure over-fitting did not occur. Results of the Cox model are shown in Table 1. The strongest predictors of mortality were the presence of secondary osteoporosis (HR: 1.54, 95% C.I. 0.8–2.95), and glucocorticoids (HR: 2.95, 95% C.I. 0.8–10.8). The AUC-ROC obtained by the model was 0.702, while the AUC-PR was 0.172.

### 3.2. Base Learners

Firstly, base versions of LR, DT, RF, and AdaBoost models have been considered. No further techniques of re-sampling, probability calibration, or cost-sensitive learning have been performed. The results are reported in Table 2, with results on both test and training set. The best performance was achieved by AdaBoost (AUC-ROC: 0.512), closely followed by the other models.

### 3.3. Enhanced Base Learners

All the previous base models have been combined with the different techniques described in the Methods Section.

The results for the LR model are shown in Table S1 (Supplementary Data 2). The best result has been achieved when using ADASYN with probability calibration (AUC-ROC: 0.573). Results with only over/under-sampling techniques have an AUC-ROC between 0.512 and 0.532. However, when probability calibration is applied, the general performance tended to increase (AUC-ROC between 0.510 and 0.573) with respect to the models without. Furthermore, applying cost-sensitive learning does not produce significant improvements (AUC-ROC: 0.510–0.536).

The results for the DT model are shown in Table S2 (Supplementary Data 2). The model performance is slightly worse compared to the LR case, with the highest AUC-ROC (0.541) achieved with SMOTE alone, even though the difference with the other over/under-sampling techniques was minimal (minimum AUC-ROC: 0.511). Again, the cost-sensitive learning approach slightly decreased the results (AUC-ROC between 0.507 and 0.535), while the results achieved by using probability calibration have ranked in the middle (AUC-ROC between 0.508–0.536).

The results for the RF model are shown in Table S3 (Supplementary Data 2). In this case, the highest AUC-ROC value (0.642) was reached with RUS without probability calibration. With RF models probability calibration decreased the large variability across the different sampling techniques (AUC-ROC between 0.532 and 0.606, while it was between 0.516 and 0.642 without probability calibration). Cost-sensitive learning, instead, generally decreased the overall performance (AUC-ROC: 0.506–0.534).

The results for the AdaBoost model are shown in Table S4 (Supplementary Data 2). No cost-sensitive learning was tested with AdaBoost as this classifier does not provide the “class_weight” hyper-parameter. The performance between with and without probability calibration is not significantly different (AUC-ROC: 0.518–0.541 without probability calibration, AUC-ROC: 0.519–0.543 with probability calibration), with the best result obtained with ADASYN.

**Figure 1:**
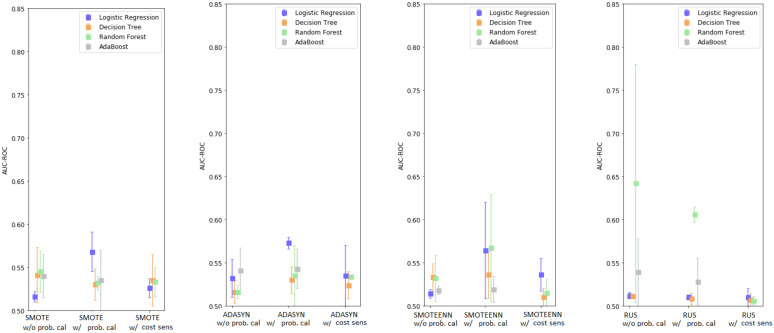
Enhanced base classifiers performance-summary.

In summary, among all the possible combinations, Random Forest showed the best performance. A statistical analysis between the best model (RF + RUS with/without probability calibration) and the best base learner shows a statistical difference only in the case with probability calibration; e.g., mean difference: 0.13, (95% C.I.: −0.00851, 0.2685), *p*-value: 0.065 (without calibration), mean difference: 0.094, (95% C.I.: 0.084, 0.1032), *p*-value: <0.001 (with calibration). Cost-sensitive learning and probability calibration, used in conjunction with resampling, generally reduced the variability of the model performance, except for LR. Using the different re-sampling techniques tended to improve the performance of some base learners; however, there was no clear winner between the different over/under-sampling techniques which did not show significant differences in general. A graphical depiction of the results is shown in Figure 1.

### 3.4. Enhanced Base Classifiers with Monte Carlo Data Augmentation

To compensate for the high imbalance of the dataset, the Monte Carlo data augmentation technique was adopted by creating synthetic data for both classes, after the adoption of the FSCA and Isolation Forest techniques. Again, all the possible combinations of models with probability calibration, cost-sensitive learning, and sampling techniques have been investigated.

The results for the LR model are shown in Table S5 (Supplementary Data 2). The highest AUC-ROC was achieved with SMOTE and ADASYN without probability calibration (AUC-ROC: 0.539). The higher performance was obtained with over/under-sampling techniques alone with AUC-ROC between 0.515 and 0.539, while probability calibration resulted in an AUC-ROC between 0.507 and 0.530, and cost-sensitive learning between 0.514 and 0.535.

The results for the DT model are shown in Table S6 (Supplementary Data 2). In this case, the adoption of sampling techniques alone provided the worst results (AUC-ROC between 0.510 and 0.519, obtained with ADASYN). Results tended to be much higher when probability calibration was adopted (AUC-ROC: 0.514–0.530), and with cost-sensitive learning (AUC-ROC: 0.521–0.529).

The results for the RF model are shown in Table S7 (Supplementary Data 2). The best performance was obtained with SMOTE combined with probability calibration (AUC-ROC: 0.535). In this case, cost-sensitive learning tended to slightly reduce the overall performance (AUC-ROC: 0.503–0.530) compared to using sampling techniques only (AUC-ROC 0.511–0.530), with the best results obtained when using probability calibration (AUC-ROC 0.511–0.535).

**Figure 2:**
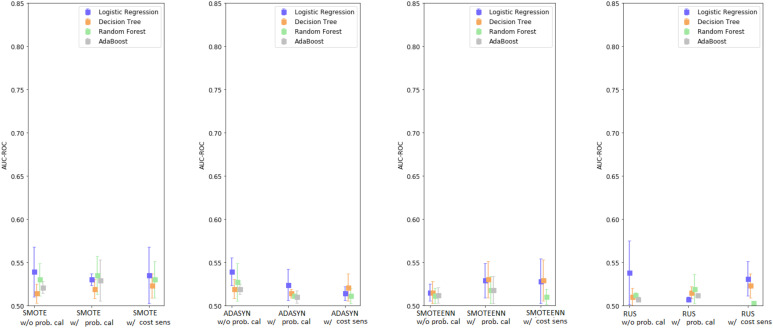
Enhanced base classifiers performance with Monte Carlo data augmentation-summary.

The results for the AdaBoost model are shown in Table S8 (Supplementary Data 2). The performance of the models without probability calibration ranged between 0.507 and 0.521, while they were between 0.510 and 0.529 (using SMOTE) with probability calibration. A graphical depiction of the results is shown in Figure 2.

Comparing Figure 1 and Figure 2, as well as Tables S1–S4 and S5–S8, it can be noticed that including synthetic data generated by the Monte Carlo process negatively affected the performance of every model. Across the different techniques, the reduction in performance for LR averaged 0.0055 points (max 0.049), for DT it was 0.004 points (max 0.027), for RF it was 0.028 points (max 0.13), and for AdaBoost it was 0.016 points (max 0.033). When comparing the best models obtained with and without data augmentation, there is a statistically significant difference in favor of the model without Monte Carlo; e.g., mean difference: 0.067, (95% C.I.: 0.04, 0.093), *p*-value: <0.001.

To investigate the validity of the synthesized samples, a simple but effective test was performed via the development of a model which can classify original vs. synthetic samples. The process for the test was as follows:

- Given the original dataset of 2291 participants, new synthetic data in the same amount have been obtained with the Monte Carlo data augmentation technique and merged into the original dataset
- The labels related to the mortality prediction problem were eliminated for this test from every subject (both original and synthetic)
- All the data belonging to the original dataset were re-labelled as class 1, while all the synthetic data were re-labelled as class 0
- The dataset was divided in 70/30 (as test set), with the 70% again split in 70/30 for training and validation purposes, with stratification being applied
- A standard RF classifier was used to discriminate between the original and the synthetic data. The model was trained on the training set with the hyper-parameters tuned on the validation set. The optimized model was finally evaluated on the test set. RF was adopted, as it was the model which presented the largest difference in performance when using the Monte Carlo technique

**Figure 3:**
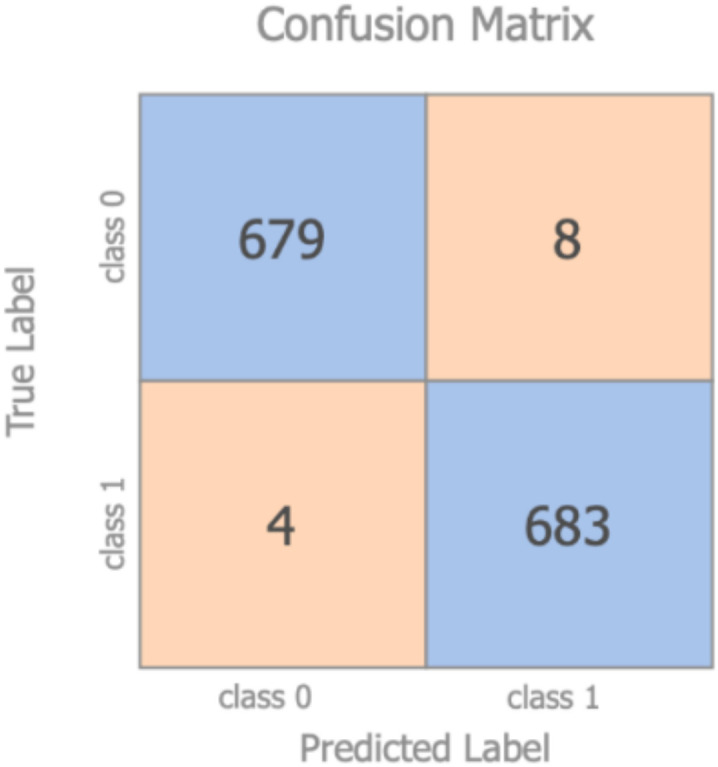
Confusion matrix–original vs. synthetic samples.

The accuracy achieved by the model was 99% (the relevant confusion matrix is shown in Figure 3), indicating the ease for the classifier to discriminate between original and synthetic samples.

## 4. Discussion

All-cause mortality prediction is significant for the development of personalized risk management. ML has shown in literature the possibility to outperform existing models for predicting all-cause mortality not only in subjects with specific health conditions but also in prospective studies [36]. This work developed an ML model able to predict a two to seven year all-cause mortality in a cohort of healthy older adults based on features covering anthropometric variables, physical and lab examinations, questionnaires and lifestyles, and wearable data collected in free-living settings, and provided a comprehensive comparison on the impact shown by the standard techniques for imbalanced datasets on the overall model. For this purpose, several ML techniques (e.g., data augmentation, resampling, and so on) were investigated. A summary of the main results shown in Tables S1–S8, limited to AUC-ROC, AUC-PR, precision, and recall, is illustrated in Table 3 to better support the reader.

In terms of data augmentation, a Monte Carlo approach proposed in [55] has been investigated for its ease-of-use and optimal results achieved in other contexts (i.e., industrial processes). However, with the present dataset, this approach delivered unsatisfactory results, consistently underperforming when compared to models without data augmentation. This was due to the creation of new synthetic patient records which could be easily distinguishable from real patients’ data, as proven in the present investigation. Those synthetic records were easily distinguishable because of the approach used for their generation in which values were generated randomly for each feature and with this process repeated independently for each feature. This was a clear signal of the limitations of this Monte Carlo data augmentation technique despite the attention paid in making sure the synthetic data met the criteria of the original dataset (e.g., by forcing categorical variables). Despite this technique being introduced in the literature as a method for augmenting data in predictive fault detection, it seems evident this Monte Carlo technique may not be feasible for health-related datasets of this type.

For this reason, it may be useful to investigate the adoption of other data augmentation techniques in health-related datasets, such as Bayesian networks, a mixture of product of multinomials, or Generative Adversarial Networks (GAN) [64]. The generation of realistic synthetic EHR patient data is still an open question which can potentially mitigate the challenges associated with limited sample sizes [65]. Yet this is an under investigated area in the field.

Moreover, when dealing with a high-class imbalanced dataset, different techniques at data-level (e.g., over/under-sampling) or algorithm-level (e.g., cost-sensitive) are popular in addressing the imbalance. The results obtained show that, as expected, those techniques provide an overall improvement in the performance when compared to the base learners’ models. However, the obtained results did not show significant differences across the different techniques, thus confirming the findings shown in [66], namely that there is essentially no performance difference between the over-sampling techniques. As indicated in [67], which compared 85 algorithms carried out on 104 datasets, no sampling technique provided consistently superior performance on each model, therefore indicating that sampling algorithms need to be designed to accommodate the intrinsic characteristics of the dataset under consideration, and that model selection and the sampler’s hyper-parameters selection are a challenging dataset-dependent process.

This investigation showed that, for the dataset under consideration, the best results were achieved with RUS in conjunction with RF (either with or without probability calibration). However, while including probability calibration slightly reduced the average performance, it increased the model robustness, as indicated by the lower 95% confidence intervals, and provided a better AUC-PR score. The 95% C.I. of the model without probability calibration were large (0.504–0.781), highlighting a possible concern in ML models associated with a potential lack of reproducibility. This may be even more evident in small size datasets, as those results could be more significantly affected by a particular training/test data split. Therefore, this still presents a challenge for the adoption of ML models into clinical practice. Moreover, the large 95% C.I. shows that the ML model could potentially outperform the Cox epidemiological model used as a baseline; however, on average, Cox still performed better. Those results confirmed the findings reported in [68], e.g., that random forests provided good results, comparable to expert-based models, but still did not outperform Cox models. This is despite their inherent ability to accommodate nonlinearities and interactions, even if trained on a large dataset of a sample size of over 80,000 patients. Therefore, more complex approaches, such as ensemble models, should be investigated in the future to further improve the performance when dealing with this highly imbalanced small size dataset.

However, RUS + RF without probability calibration presented an AUC-PR of 0.337 and its variant with probability calibration an AUC-PR of 0.467, which were two to three times larger than the baseline obtained with the standard Cox model (AUC-PR: 0.172). Given the similar AUC-ROC performance between the models, the fact that ML provides better AUC-PR compared to the standard Cox model indicates that ML shows a lower misclassification of the examples in the minority positive class compared to baseline [69] (e.g., lower false negatives), thus highlighting the benefits of using ML compared to epidemiological models. Moreover, as is evident in Table S3, RUS + RF with probability calibration showed a high recall (>0.87) with a low precision (0.05), indicating that the number of true positives detected by the model is very high and the number of false positives is significantly larger compared to the number of false negatives. Namely, the model correctly identifies >87% of the dead participants in the dataset as such, while presenting a large number of false positives (e.g., subjects predicted to die while actually being still alive). As in clinical practice, the main goal is to detect as accurately as possible “high mortality risk” subjects, so minimizing the false negatives is more important than minimizing the false positives (especially when dealing with imbalanced data); therefore, a high recall is essential and this is achieved by the developed model. While these results are promising, further work is still required to improve the overall precision of the model for it to be acceptable for clinical practice.

The presented results are comparable to the outcomes reported in similar studies ([70], for example, with the best mortality prediction model showing an AUC-ROC of 0.69, 95% CI: 0.61–0.76, in a sepsis cohort of 2510 patients with 11.5% positive cases). However, as indicated in another example [71], investigating 90-days mortality prediction models in a cohort of 800 patients (8% positive class), AUC-ROC can portray an overly-optimistic performance of a classifier risk score when applied to imbalanced data and AUC-PR provides better insight about the performance of a classifier by focusing on the minority class. Even though this trend is well reported in the clinical literature, AUC-ROC is still the main metric generally adopted. For this reason, the present study presents both AUC-ROC and AUC-PR. When comparing [71] with the results achieved in the present analysis, our model can provide comparable if not better results despite dealing with an even more imbalanced dataset (AUC-PR: 0.467 vs. 0.43 in [71]).

It is worth highlighting that most of the models considered in this study can only provide binary indications on the mortality prediction of the subjects under test, and behave as a black-box, thus without indicating the possible rationale behind those indications. The lack of interpretability of the model could represent a limiting factor to its adoption in clinical settings, as clinicians are focusing also on model interpretation, which is critical to obtain domain knowledge from the computational analysis. However, decision tree-based models can also provide domain knowledge which is easy to understand for clinicians (unlike ensembles, for example), but compared to the other considered models they are generally affected by lower performance (as shown in Tables S1–S8). On the other hand, Cox models can provide this domain knowledge despite the limitations described before. As interpretability is now becoming a significant factor to take into account when developing predictive modelling in healthcare [72], it should be considered for future research in the area. A recent example in the field is represented by [73], which highlighted how explainable ML could generate explicit knowledge of how models make their predictions, and that ML-based models can perform at least as good as classical Cox models and in some cases even better than them

While there is a large number of papers comparing the predictive performance of ML models in literature, the strength of the study is, in particular, in the breadth of techniques taken into consideration, such as over/under-sampling, cost-sensitive learning, data augmentation, probability calibration, etc., and in the methodology adopted, which guarantees a fair comparison between the models. None of the developed models was affected by overfitting, as is evident from the limited difference in performance between training and testing results. Even though past studies have looked at comparing different approaches in the same technique (for example, comparing several data augmentation approaches with each other [64], or different over/under-sampling techniques [67]), very few studies have actually investigated the impact that the combination of these techniques (e.g., oversampling with probability calibration and data augmentation) could have on the mode’s performance. However, despite the numerous techniques adopted to represent over/under-sampling in this study, those are still a fraction compared to the huge number of sampling variants currently proposed in the literature [67], and further work will be required to also include those methodologies.

Moreover, while a number of recent studies have investigated the comparison of ML models in mortality prediction [74,75,76], those works have only taken into consideration acute patients’ data samples in ICUs and not the more complex scenario of mortality prediction in the general population. Moreover, [74,75,76] have not investigated the impact that several techniques for handling imbalanced datasets had on the overall results. This study, therefore, provides an empirical baseline for the field and provides possible indications for future research in the area of ML applied to the problem of mortality prediction in the general population, such as the generation of realistic synthetic EHR patient data, improvements of model robustness for an effective adoption into clinical practice, model interpretability, and the development of more complex models (i.e., ensembles) able to outperform expert-based models.

Finally, another limitation of this study is that those considerations are obtained from a single study dataset, thus replication in other settings (i.e., ICU) is required to further prove the generalizability of the findings. Likewise, the dataset was collected from older adults in Sweden, therefore it is unclear how generalizable those results are to other populations worldwide.

## 5. Conclusions

Ageing is a global phenomenon of much relevance to our rapidly changing society, and all-cause mortality prediction represents an important tool for essential healthcare resource allocation and risk management. In this paper, results on the development of a ML model for all-cause mortality prediction in a cohort of healthy older adults are reported. The analysis is based on features covering anthropometric variables, physical and lab examinations, questionnaires, and lifestyles, as well as wearable data collected in free-living settings, which are not generally included in survival analysis. Several techniques, such as feature engineering, feature selection, data augmentation, over/under-sampling, probability calibration, and a cost-sensitive approach were discussed and investigated for this purpose. The models were also compared to a Cox epidemiological model as a reference. Considerations were drawn on different aspects related to the performance of the data augmentation technique, resampling and models selected in this paper for investigation. It was demonstrated that ML models could provide comparable results to standard epidemiological models while being completely data-driven and disease-agnostic; however, further testing is required to significantly improve the model performance and robustness. Future steps include the investigation of ensemble models that could tackle the highly imbalanced small sample size problem of the present dataset, as well as the application to disease-specific sub-cohorts.

## Figures and Tables

**Table 1:** Cox Model.

Variables	Hazard Ratio (HR)	HR 95% C.I.	*p*-Value
Sex	0.47	0.27–0.83	0.01
HDL Cholesterol	0.88	0.83–0.93	<0.005
Rheumatoid arthritis	0.20	0.02–2.43	0.21
Total T-score	0.77	0.62–0.96	0.02
Secondary osteoporosis	1.54	0.8–2.95	0.2
Glucocorticoids	2.95	0.8–10.8	0.1
Parent Fractured Hip	0.68	0.31–1.5	0.34
Sway trace length (no vision)	1	1.0–1.0	0.02
IPAQ MET-min per week	1	1.0–1.0	0.01

**Table 2:** Base classifiers performance.

MODEL		AUC-ROC	AUC-PR	BRIER SCORE	F1 SCORE	ACCURACY	RECALL	PRECISION
**LR**	Test	0.509	0.222	0.746	0.048	0.954	0.036	0.072
		(0.500–0.527)	(0.020–0.520)	(0.741–0.753)	(0.000–0.095)	(0.945–0.959)	(0.000–0.071)	(0.000–0.143)
	Train	0.568	0.280	0.543	0.079	0.957	0.536	0.043
		(0.534–0.601)	(0.251–0.297)	(0.543–0.544)	(0.072–0.087)	(0.956–0.957)	(0.429–0.679)	(0.038–0.048)
**DT**	Test	0.511	0.094	0.596	0.068	0.904	0.095	0.056
		(0.507–0.514)	(0.020–0.148)	(0.566–0.634)	(0.025–0.120)	(0.856–0.930)	(0.000–0.143)	(0.000–0.096)
	Train	0.522	0.104	0.587	0.079	0.913	0.093	0.076
		(0.518–0.527)	(0.095–0.121)	(0.577–0.593)	(0.069–0.086)	(0.907–0.918)	(0.079–0.112)	(0.059–0.092)
**RF**	Test	0.509	0.187	0.044	0.040	0.956	0.030	0.052
		(0.508–0.509)	(0.020–0.520)	(0.041–0.045)	(0.000–0.080)	(0.955–0.959)	(0.000–0.060)	(0.000–0.104)
	Train	0.570	0.106	0.044	0.028	0.956	0.015	0.167
		(0.551–0.590)	(0.092–0.117)	(0.043–0.045)	(0.010–0.046)	(0.955–0.957)	(0.000–0.031)	(0.000–0.300)
**ADABOOST**	Test	**0.512**	0.054	0.547	0.020	0.953	0.012	0.056
		**(0.510–0.514)**	(0.020–0.121)	(0.547–0.548)	(0.000–0.059)	(0.952–0.953)	(0.000–0.036)	(0.000–0.167)
	Train	0.582	0.113	0.549	0.056	0.951	0.036	0.138
		(0.553–0.611)	(0.068–0.138)	(0.546–0.552)	(0.039–0.074)	(0.949–0.953)	(0.015–0.047)	(0.040–0.196)

LR: Logistic Regression, DT: Decision Tree, RF: Random Forest, AdaBoost: Adaptive Boosting, AUC-ROC: Area Under the Curve–Receiver Operative Curve, AUC-PR: Area Under the Curve–Precision-Recall Curve. Results obtained on test and training sets. For each metric mean value and its 95% confidence interval are reported. Bold: the model with best results as done before.

**Table 3:** Enhanced base learners performance (selected cases summary).

Model	AUC-ROC	AUC-PR	Precision	Recall
**LR W/ ADASYN** **W/ PROB. CAL.**	0.573 (0.566–0.582)	0.311 (0.288–0.339)	0.055 (0.054–0.056)	0.548 (0.500–0.596)
**DT W/ SMOTE** **W/O PROB. CAL.**	0.541 (0.509–0.567)	0.317 (0.123–0.425)	0.045 (0.033–0.052)	0.572 (0.179–0.78)
**RF W/ RUS** **W/O PROB. CAL.**	**0.642** **(0.504–0.781)**	**0.337** **(0.121–0.553)**	**0.068** **(0.029–0.107)**	**0.590** **(0.179–1.00)**
**RF W/ RUS** **W/ PROB. CAL.**	**0.606** **(0.597–0.615)**	**0.467** **(0.441–0.493)**	**0.053** **(0.053–0.054)**	**0.875** **(0.821–0.929)**
**ADABOOST W/ ADASYN** **W/ PROB. CAL.**	0.543 (0.520–0.582)	0.364 (0.300–0.419)	0.047 (0.044–0.053)	0.667 (0.536–0.769)
**LR W/ SMOTE** **W/O PROB. CAL. W/ D.A.**	0.539 (0.510–0.559)	0.262 (0.247–0.288)	0.049 (0.045–0.056)	0.453 (0.429–0.500)
**LR W/ ADASYN** **W/O PROB. CAL. W/ D.A.**	0.539 (0.523–0.555)	0.273 (0.260–0.288)	0.049 (0.046–0.055)	0.476 (0.429–0.500)
**DT W/ SMOTEENN** **W/ PROB. CAL. W/ D.A.**	0.530 (0.509–0.560)	0.448 (0.371–0.503)	0.044 (0.040–0.047)	0.845 (0.679–0.964)
**RF W/ SMOTE** **W/ PROB. CAL. W D.A.**	0.535 (0.513–0.557)	0.280 (0.176–0.336)	0.050 (0.038–0.065)	0.488 (0.286–0.607)
**ADABOOST W/ SMOTE** **W/ PROB. CAL. W/ D.A.**	0.529 (0.505–0.554)	0.172 (0.074–0.255)	0.046 (0.039–0.052)	0.476 (0.286–0.786)

LR: Logistic Regression, DT: Decision Tree, RF: Random Forest, AdaBoost: Adaptive Boosting, AUC-ROC: Area Under the Curve–Receiver Operative Curve, AUC-PR: Area Under the Curve–Precision-Recall Curve, SMOTE: Synthetic Minority Over-sampling Technique, ADASYN: Adaptive Synthetic, RUS: Random under-Sampling, SMOTEENN: Synthetic Minority Over-sampling Technique Edited Nearest Neighbor, w/: with, w/o: without, prob.cal.: probability calibration, D.A: data augmentation. Only results obtained on test set reported. For each metric mean value and its 95% confidence interval are reported. Bold: the model with best results as done before.

## Data Availability

The datasets analyzed during the current study are not publicly available due ethical and national regulations, but are available from the corresponding author on reasonable request.

## Supplementary Materials

The following are available online at https://www.mdpi.com/article/10.3390/ijerph182312806/s1, Figure S1A: Forward Selection Component Analysis pseudo-code. Figure S2A: Monte Carlo data augmentation pseudo-code. Table S1: Enhanced logistic regression performance. Table S2: Enhanced decision tree performance. Table S3: Enhanced random forest performance. Table S4: Enhanced Adaptive Boosting Performance. Table S5: Enhanced Logistic Regression with Data Augmentation Performance. Table S6: Enhanced Decision Tree with Data Augmentation Performance. Table S7: Enhanced Random Forest with Data Augmentation Performance. Table S8: Enhanced Adaptive Boosting with Data Augmentation Performance.

## Abbreviations

AdaBoost	Adaptive Boosting
ADASYN	Adaptive Synthetic
APACHE	Acute Physiology and Chronic Health Evaluation
AUC-PR	Area Under the Curve–Precision Recall
AUC-ROC	Area Under the Curve–Receiver Operating Characteristic
AUDIT-C	Alcohol Use Disorders Identification Test
BMI	Body Mass Index
CI	Confidence Interval
CV	Cross-Validation
DT	Decision Tree
DXA	X-ray Absorptiometry
EHR	Electronic Health Records
EuroSCORE	European System for Cardiac Operative Risk Evaluation
FI	Frailty Index
FSCA	Forward Selection Component Analysis
GDS	Geriatric Depression Scale
HAI	Healthy Ageing Initiative
HR	Hazard Ratio
ICU	Intensive Care Unit
IPAQ	International Physical Activity Questionnaire
LR	Logistic Regression
MI	Mortality Index
ML	Machine Learning
MMT	Manual Muscle Test
MPM	Mortality Probability Model
pQCT	Computed Tomography
RF	Random Forest
RUS	Random UnderSampling
SAPS	Simplified Acute Physiology Score
SMOTE	Synthetic Minority Oversampling Technique
SMOTEENN	SMOTE and Edited Nearest Neighbours
TUG	Time Up and Go
VIF	Variance Inflation Factor

## References

[1] Wittenberg R.D., Comas-Herrera A., Pickard L., Hancock R. (2004). Future Demand for Long-Term Care in the UK: A Summary of Projections of Long-Term Care Finance for Older People to 2051.

[2] Eurostat Ageing Europe—Statistics on Population Developments. https://ec.europa.eu/eurostat/statistics-explained/index.php?title=Ageing_Europe_-_statistics_on_population_developments#Older_people_.E2.80.94_population_overview.

[3] de Munter L., Polinder S., Lansink K.W.W., Cnossen M.C., Steyerberg E.W., de Jongh M.A.C. (2017). Mortality prediction models in the general trauma population: A systematic review. Injury.

[4] Keuning B.E., Kaufmann T., Wiersema R., Granholm A., Pettila V., Moller M.H., Christiansen C.F., Castela Forte J., Keus F., Pleijhuis R.G. (2020). Mortality prediction models in the adult critically ill: A scoping review. Acta Anaesthesiol. Scand..

[5] Xie J., Su B., Li C., Lin K., Li H., Hu Y., Kong G. (2017). A review of modeling methods for predicting in-hospital mortality of patients in intensive care unit. J. Emerg. Crit. Care. Med..

[6] Tosato M., Zamboni V., Ferrini A., Cesari M. (2007). The aging process and potential interventions to extend life expectancy. Clin. Interv. Aging.

[7] National Research Council (US) (2001). Panel on a Research Agenda and New Data for an Aging World Preparing for an Aging World: The Case for Cross-National Research.

[8] Yourman L.C., Lee S.J., Schonberg M.A., Widera E.W., Smith A.K. (2012). Prognostic indices for older adults: A systematic review. JAMA.

[9] Knaus W.A., Zimmerman J.E., Wagner D.P., Draper E.A., Lawrence D.E. (1981). APACHE-acute physiology and chronic health evaluation: A physiologically based classification system. Crit. Care Med..

[10] Le Gall J.R., Loirat P., Alperovitch A., Glaser P., Granthil C., Mathieu D., Mercier P., Thomas R., Villers D. (1984). A simplified acute physiology score for ICU patients. Crit. Care Med..

[11] Lemeshow S., Teres D., Avrunin J.S., Pastides H. (1987). A comparison of methods to predict mortality of intensive care unit patients. Crit. Care Med..

[12] Nashef S.A., Roques F., Michel P., Gauducheau E., Lemeshow S., Salamon R. (1999). European system for cardiac operative risk evaluation (EuroSCORE). Eur. J. Cardiothorac. Surg..

[13] Spector W.D., Takada H.A. (1991). Characteristics of nursing homes that affect resident outcomes. J. Aging Health.

[14] Graf C. (2008). The lawton instrumental activities of daily living scale. AJN.

[15] Walsh M., O’Flynn B., O’Mathuna C., Hickey A., Kellett J. (2013). Correlating average cumulative movement and Barthel Index in acute elderly care. International Joint Conference Ambient Intelligence.

[16] Higuchi S., Kabeya Y., Matsushita K., Taguchi H., Ishiguro H., Kohshoh H., Yoshino H. (2016). Barthel index as a predictor of 1-year mortality in very elderly patients who underwent percutaneous coronary intervention for acute coronary syndrome: Better activities of daily living, longer life. Clin. Cardiol..

[17] Torsney K.M., Romero-Ortuno R. (2018). The Clinical Frailty Score predicts inpatient mortality in older hospitalized patients with idiopathic Parkinson’s disease. J. R Coll. Physicians Edinb..

[18] Moreno R.P. (2008). Outcome prediction in intensive care: Why we need to reinvent the wheel. Curr. Opin. Crit. Care.

[19] Booth H., Tickle L. (2008). Mortality modelling and forecasting: A review of methods. Ann. Actuar. Sci..

[20] Pitacco E., Denuit M., Haberman S., Olivieri A. (2009). Modelling Longevity Dynamics for Pensions and Annuity Business.

[21] Richman R., Wuthrich W. (2018). A neural network extension of the Lee-Carter model to multiple populations. Ann. Actuar. Sci..

[22] Levantesi S., Pizzorusso V. (2019). Application of machine learning to mortality modeling and forecasting. Risks.

[23] Gulshan V., Peng L., Coram M., Stumpe M.C., Wu D., Narayanaswamy A., Venugopalan S., Widner K., Madams T., Cuadros J. (2016). Development and validation of a deep learning algorithm for detection of diabetic retinopathy in retinal fundus photographs. JAMA.

[24] Weng S.F., Reps J., Kai J., Garibaldi J.M., Qureshi N. (2017). Can machine-learning improve cardiovascular risk prediction using routine clinical data?. PLoS ONE.

[25] Komaris D.S., Perez-Valero E., Jordan L., Barton J., Hennessy L., O’Flynn B., Tedesco S. (2019). Predicting three-dimensional ground reaction forces in running by using artificial neural networks and lower body kinematics. IEEE Access.

[26] Tedesco S., Crowe C., Ryan A., Sica M., Scheurer S., Clifford A.M., Brown K.N., O’Flynn B. (2020). Motion sensors-based machine learning approach for the identification of anterior cruciate ligament gait patterns in on-the-field activities in rugby players. Sensors.

[27] Parikh R.B., Manz C., Chivers C., Harkness Regli S., Braun J., Draugelis M.E., Schuchter L.M., Shulman L.N., Navathe A.S., Patel M.S. (2019). Machine learning approaches to predict 6-month mortality among patients with cancer. JAMA Netw. Open.

[28] Metsker O., Sikorsky S., Yakovlev A., Kovalchuk S. (2018). Dynamic mortality prediction using machine learning techniques for acute cardiovascular. Procedia Comput. Sci..

[29] Kang M.W., Kim J., Kim D.K., Oh K.-H., Joo K.W., Kim Y.S., Han S.S. (2020). Machine learning algorithm to predict mortality in patients undergoing continuous renal replacement therapy. Crit. Care.

[30] Du X., Min J., Shah C.P., Bishnoi R., Hogan W.R., Lemas D.J. (2020). Predicting in-hospital mortality of patients with febrile neutropenia using machine learning models. Int. J. Med. Inform..

[31] Moll M., Qiao D., Regan E.A., Hunninghake G.M., Make B.J., Tal-Singer R., McGeachie M.J., Castaldi P.J., San Jose Estepar R., Washko G.R. (2020). Machine learning and prediction of all-cause mortality in COPD. Chest.

[32] Lund J.L., Kuo T.-M., Brookhart M.A., Meyer A.M., Dalton A.F., Kistler C.E., Wheeler S.B., Lewis C.L. (2019). Development and validation of a 5-year mortality prediction model using regularized regression and Medicare data. Pharmacoepidemiol. Drug Saf..

[33] Meyer A., Zverinski D., Pfahringer B., Kempfert J., Kuehne T., Sundermann S.H., Stamm C., Hofmann T., Falk V., Eickhoff C. (2018). Machine learning for real-time prediction of complications in critical care: A retrospective study. Lancet Respir. Med..

[34] Shouval R., Labopin M., Bondi O., Mishan-Shamay H., Shimoni A., Ciceri F., Esteve J., Giebel S., Gorin N.C., Schmid C. (2015). Prediction of allogeneic hematopoietic stem-cell transplantation mortality 100 days after transplantation using a machine learning algorithm: A European group for blood and marrow transplantation acute leukemia working party retrospective data mining study. J. Clin. Oncol..

[35] Liao J., Muniz-Terrera G., Scholes S., Hao Y., Chen Y.M. (2018). Lifestyle index for mortality prediction using multiple ageing cohorts in the USA, UK, and Europe. Sci. Rep..

[36] Weng S.F., Vaz L., Qureshi N., Kai J. (2019). Prediction of premature all-cause mortality: A prospective general population cohort study comparing machine-learning and standard epidemiological approaches. PLoS ONE.

[37] Clift A.K., Le Lannou E., Tighe C.P., Shah S.S., Beatty M., Hyvarinen A., Lane S.J., Strauss T., Dunn D.D., Lu J. (2021). Development and validation of risk scores for all-cause mortality for the purposes of a smartphone-based “general health score” application: A prospective cohort study using the UK Biobank. JMIR Mhealth Uhealth.

[38] Healthy Ageing Initiative. https://www.healthyageinginitiative.com/.

[39] Ballin M., Nordstrom P., Niklasson J., Alamaki A., Condell J., Tedesco S., Nordstrom A. (2020). Daily step count and incident diabetes in community-dwelling 70-years-olds: A prospective cohort study. BMC Public Health.

[40] ActiGraph. https://actigraphcorp.com/.

[41] Burke W.J., Roccaforte W.H., Wengel S.P. (1991). The short form of the geriatric depression scale: A comparison with the 30-item form. J. Geriatr. Psychiatry Neurol..

[42] AUDIT-C Score. https://www.mdcalc.com/audit-c-alcohol-use.

[43] Craig C.L., Marshall A.L., Sjöström M., Bauman A.E., Booth M.L., Ainsworth B.E., Pratt M., Ekelund U., Yngve A., Sallis J.F. (2003). International physical activity questionnaire: 12-country reliability and validity. Med. Sci. Sports Exerc..

[44] GAITRite. https://www.gaitrite.com/.

[45] Burnham J.P., Lu C., Yaeger L.H., Bailey T.C., Kollef M.H. (2018). Using wearable technology to predict health outcomes: A literature review. J. Am. Med. Inform. Assoc..

[46] Haixiang G., Yijiang L., Shang J., Mingyun G., Yuanyue H., Bing G. (2017). Learning from class-imbalanced data: Review of methods and applications. Expert Syst. Appl..

[47] Dent E., Kowal P., Hoogendijk E.O. (2006). Frailty measurement in research and clinical practice: A review. Eur. J. Intern. Med..

[48] Williams D.M., Jylhava J., Pedersen N.L., Hagg S. (2019). A frailty index for UK biobank participants. J. Gerontol. A Biol. Sci. Med. Sci..

[49] Kim N.H., Cho H.J., Kim S., Seo J.H., Lee H.J., Yu J.H., Chung H.S., Yoo H.J., Seo J.A., Kim S.G. (2016). Predictive mortality index for community-dwelling elderly Koreans. Medicine.

[50] Remeseiro B., Bolon-Canedo V. (2019). A review of feature selection methods in medical applications. Comput. Biol. Med..

[51] Puggini L., McLoone S. (2017). Forward selection component analysis: Algorithms and applications. IEEE Trans. Pattern Anal. Mach. Intell..

[52] Puggini L., McLoone S. (2016). Feature selection for anomaly detection using optical emission spectroscopy. IFAC PapersOnLine.

[53] Ding Z., Fei M. (2013). An anomaly detection approach based on isolation forest algorithm for streaming data using sliding window. IFAC Proc. Vol..

[54] Xiao C., Choi E., Sun J. (2018). Opportunities and challenges in developing deep learning models using electronic health records data: A systematic review. J. Am. Med. Inform. Assoc..

[55] Parente A.P., de Souza M.B., Valdman A., Mattos R.O. (2019). Folly data augmentation applied to machine learning-based monitoring of a pulp and paper process. Processes.

[56] Chawla N.V., Bowyer K.W., Hall L.O., Kegelmeyer W.P. (2002). SMOTE: Synthetic minority oversampling technique. J. Artif. Intell. Res..

[57] He H., Bai Y., Garcia E.A., Li S. ADASYN: Adaptive synthetic sampling approach for imbalanced learning. Proceedings of the IEEE International Joint Conference on Neural Networks (IJCNN).

[58] He H., Garcia E.A. (2009). Learning from imbalanced data. IEEE Trans. Knowl. Data Eng..

[59] Batista G.E., Prati R.C., Monard M.C. (2004). A study of the behavior of several methods for balancing machine learning training data. ACM SIGKDD Explor. Newsl..

[60] Thai-Nghe N., Gantner Z., Schmidt-Thieme L. Cost-sensitive learning methods for imbalanced data. Proceedings of the IEEE International Joint Conference on Neural Networks (IJCNN).

[61] Hsu J.L., Hung P.C., Lin H.Y., Hsieh C.H. (2015). Applying under-sampling techniques and cost-sensitive learning methods on risk assessment of breast cancer. J. Med. Syst..

[62] Wallace B.C., Dahabreh I.J. Class probability estimates are unreliable for imbalanced data (and how to fix them). Proceedings of the IEEE 12th International Conference Data Mining.

[63] Pozzolo A.D., Caelen O., Johnson R.A., Bontempi G. Calibrating probability with undersampling for unbalanced classification. Proceedings of the IEEE Symposium Series Computational Intelligence.

[64] Goncalves A., Ray P., Soper B., Stevens J., Coyle L., Sales A.P. (2020). Generation and evaluation of synthetic patient data. BMC Med. Res. Methodol..

[65] Fowler E.E., Berglund A., Schell M.J., Sellers T.A., Eschrich S.E., Heine J. (2020). Empirically-derived synthetic populations to mitigate small sample sizes. J. Biomed. Inform..

[66] Leevy J.L., Khoshgoftaar T.M., Bauder R.A., Seliya N. (2018). A survey on addressing high-class imbalance in big data. J. Big Data.

[67] Kovacs G. (2019). An empirical comparison and evaluation of minority oversampling techniques on a large number of imbalanced datasets. Appl. Soft. Comput..

[68] Steele A.J., Denaxas S.C., Shaha A.D., Hemingway H., Luscombe N.M. (2018). Machine learning models in electronic health records can outperform conventional survival models for predicting patient mortality in coronary artery disease. PLoS ONE.

[69] Davis J., Goadrich M. The relationship between precision-recall and ROC curves. Proceedings of the 23rd International Conference on Machine Learning.

[70] Rodríguez A., Mendoza D., Ascuntar J., Jaimes F. (2021). Supervised classification techniques for prediction of mortality in adult patients with sepsis. Am. J. Emerg. Med..

[71] Movahedi F., Padman R., Antaki J.F. (2020). Limitations of ROC on imbalanced data: Evaluation of LVAD mortality risk scores. arXiv.

[72] Stiglic G., Kocbek P., Fijacko N., Zitnik M., Verbert K., Cilar L. (2020). Interpretability of machine learning based prediction models in healthcare. WIREs Data Min. Knowl. Discov..

[73] Moncada-Torres A., van Maaren M.C., Hendriks M.P., Siesling S., Geleijnse G. (2021). Explainable machine learning can outperform Cox regression predictions and provide insights in breast cancer survival. Sci. Rep..

[74] Subudhi S., Verma A., Patel A.B., Hardin C.C., Khandekar M.J., Lee H., McEvoy D., Stylianopoulos T., Munn L.L., Dutta S. (2021). Comparing machine learning algorithms for predicting ICU admission and mortality in COVID-19. NPJ Digit. Med..

[75] Yun K., Oh J., Hong T.H., Kim E.Y. (2021). Prediction of mortality in surgical intensive care unit patient using machine learning algorithms. Front. Med..

[76] Servia L., Montserrat N., Badia M., Llompart-Pou J.A., Barea-Mendoza J.A., Chico-Fernandez M., Sanchez-Casado M., Jimenez J.M., Mayor D.M., Trujillano J. (2020). Machine learning techniques for mortality prediction in critical traumatic patients: Anatomic physiologic variables from the RETRAUCI study. BMC Med. Res. Methodol..

