# Supplementary material for: Comparison of Machine Learning Techniques for Mortality Prediction in a Prospective Cohort of Older Adults
Source: Int J Environ Res Public Health. 2021 Dec 4;18(23):12806. doi: 10.3390/ijerph182312806 (PMC8657506; doi:10.3390/ijerph182312806)
Supplement: Supplementary file 1 [file ijerph-18-12806-s001.zip › SupplementaryData1.pdf]

Algorithm 1 Forward Selection Component Analysis

$Z_k = \mathbf{FSCA}(X, K)$

1. Start with the full data $X = (x_1, \dots, x_p)$ and K the number of variables to select. Initialize $Z_0 = \emptyset$ and $k = 0$.
2. Define Z^v_{k+1} as the matrix Z_k with the addition of the variable x_v.
3. Define Z_{k+1} as:

$$argmin_v \| X - Z^v_{k+1}({Z^v_{k+1}}^T Z^v_{k+1})^{-1} {Z^v_{k+1}}^T X \|_2$$

4. Update $k = k + 1$
5. If $k \leq K$ return to step 3. Otherwise output Z_K, the set of selected variables.

Figure S1.A Forward Selection Component Analysis pseudo-code

Algorithm 2 Monte Carlo data augmentation

Require: Set n as the number of new synthetic samples to generate. Define D as real process data matrix with length l.

Ensure: Matrix P containing n new artificial samples.

Generate n pseudo-random numbers, ranging between 0 and 1 and create $rand$ vector to store them.

Define D_{min} and D_{max} as vectors containing the minimum and maximum values of each feature, respectively.

For each feature column, compute its min and max value and store them in D_{min} and D_{max}.

Compute $P = D_{min} + (D_{max} - D_{min}) \cdot rand$, simulated process data matrix.

Estimate the Sqr Euclidean Distances between each real data and each simulated data as:

$$Dist_{kj} = \sum \frac{(D_{j,i} - P_k)^2}{D_{jMax}}; \quad k = 1:n \quad i = 1:l \quad j = 1:l$$

Define K as number of neighbours.

For each P_k new sample, apply K Nearest Neighbours algorithm by means of computed $Dist_{kj}$, to perform class prediction and assign class label.

return P

Figure S2.A Monte Carlo data augmentation pseudo-code
