# Supplementary material for: Comparison of Machine Learning Techniques for Mortality Prediction in a Prospective Cohort of Older Adults
Source: Int J Environ Res Public Health. 2021 Dec 4;18(23):12806. doi: 10.3390/ijerph182312806 (PMC8657506; doi:10.3390/ijerph182312806)
Supplement: Supplementary file 1 [file ijerph-18-12806-s001.zip › SupplementaryData2.pdf]

TABLE S1 ENHANCED LOGISTIC REGRESSION PERFORMANCE

MODEL		AUC-ROC	AUC-PR	BRIER SCORE	F1 SCORE	ACCURACY	RECALL	PRECISION
**LR W/ SMOTE W/O PROB. CAL.**	Test	0.516 (0.510- 0.519)	0.251 (0.184 - 0.308)	0.411 (0.381 - 0.442)	0.080 (0.058 - 0.098)	0.589 (0.557 - 0.625)	0.436 (0.321 - 0.550)	0.044 (0.033 - 0.055)
	Train	0.574 (0.559 - 0.590)	0.087 (0.071 - 0.103)	0.384 (0.363 - 0.402)	0.099 (0.095 - 0.103)	0.616 (0.602 - 0.632)	0.497 (0.489 - 0.504)	0.055 (0.053 - 0.059)
**LR W/ ADASYN W/O PROB. CAL.**	Test	0.532 (0.510 - 0.557)	0.270 (0.219 - 0.321)	0.411 (0.387 - 0.444)	0.086 (0.068 - 0.103)	0.589 (0.553 - 0.618)	0.471 (0.382 - 0.571)	0.048 (0.038 - 0.058)
	Train	0.559 (0.546 - 0.573)	0.092 (0.073 - 0.109)	0.390 (0.376 - 0.399)	0.094 (0.091 - 0.097)	0.610 (0.601 - 0.620)	0.471 (0.447 - 0.486)	0.052 (0.051 - 0.054)
**LR W/ SMOTEENN W/O PROB. CAL.**	Test	0.514 (0.509 - 0.524)	0.296 (0.229 - 0.365)	0.499 (0.434 - 0.557)	0.080 (0.064 - 0.095)	0.501 (0.435 - 0.566)	0.528 (0.413 - 0.647)	0.043 (0.035 - 0.054)
	Train	0.568 (0.553 - 0.585)	0.083 (0.068 - 0.096)	0.522 (0.489 - 0.548)	0.091 (0.086 - 0.097)	0.478 (0.454 - 0.505)	0.609 (0.577 - 0.640)	0.049 (0.047 - 0.053)
**LR W/ RUS W/O PROB. CAL.**	Test	0.512 (0.508 - 0.516)	0.270 (0.220 - 0.316)	0.477 (0.443 - 0.510)	0.076 (0.063 - 0.091)	0.523 (0.487 - 0.562)	0.479 (0.364 - 0.564)	0.041 (0.033 - 0.051)
	Train	0.550 (0.537 - 0.566)	0.084 (0.069 - 0.098)	0.483 (0.468 - 0.499)	0.094 (0.090 - 0.098)	0.517 (0.501 - 0.530)	0.582 (0.574 - 0.591)	0.051 (0.048 - 0.054)
**LR W/ SMOTE W/ PROB. CAL.**	Test	0.568 (0.545 - 0.589)	0.305 (0.285 - 0.321)	0.402 (0.363 - 0.429)	0.099 (0.090 - 0.105)	0.598 (0.571 - 0.620)	0.536 (0.500 - 0.565)	0.054 (0.049 - 0.060)
	Train	0.572 (0.562 - 0.579)	0.085 (0.073 - 0.109)	0.392 (0.371 - 0.407)	0.098 (0.093 - 0.102)	0.608 (0.594 - 0.622)	0.493 (0.485 - 0.509)	0.054 (0.051 - 0.060)
**LR W/ ADASYN W/ PROB. CAL.**	Test	**0.573 (0.566 - 0.582)**	0.311 (0.288 - 0.339)	0.404 (0.368 - 0.438)	0.100 (0.097 - 0.102)	0.596 (0.562 - 0.620)	0.548 (0.500 - 0.596)	0.055 (0.054 - 0.056)
	Train	0.580 (0.570 - 0.587)	0.078 (0.062 - 0.109)	0.397 (0.388 - 0.405)	0.095 (0.093 - 0.098)	0.603 (0.595 - 0.610)	0.488 (0.471 - 0.517)	0.053 (0.050 - 0.054)
**LR W/ SMOTEENN W/ PROB. CAL.**	Test	0.564 (0.508 - 0.602)	0.349 (0.322 - 0.392)	0.498 (0.420 - 0.569)	0.095 (0.078 - 0.105)	0.502 (0.417 - 0.556)	0.631 (0.571 - 0.697)	0.051 (0.042 - 0.056)
	Train	0.567 (0.563 - 0.573)	0.085 (0.073 - 0.110)	0.536 (0.516 - 0.572)	0.088 (0.085 - 0.092)	0.464 (0.428 - 0.482)	0.609 (0.549 - 0.690)	0.047 (0.045 - 0.050)
**LR W/ RUS W/ PROB. CAL.**	Test	0.510 (0.507 - 0.512)	0.282 (0.266 - 0.300)	0.469 (0.418 - 0.502)	0.080 (0.075 - 0.083)	0.531 (0.494 - 0.582)	0.500 (0.470 - 0.536)	0.044 (0.040 - 0.046)
	Train	0.529 (0.513 - 0.544)	0.148 (0.139 - 0.163)	0.475 (0.377 - 0.526)	0.097 (0.092 - 0.102)	0.525 (0.476 - 0.623)	0.453 (0.314 - 0.564)	0.053 (0.049 - 0.058)
**COST-SENSITIVE LR W/ SMOTE**	Test	0.526 (0.515 - 0.539)	0.450 (0.371 - 0.512)	0.770 (0.559 - 0.956)	0.085 (0.078 - 0.093)	0.230 (0.047 - 0.493)	0.848 (0.714 - 0.982)	0.045 (0.041 - 0.051)
	Train	0.542 (0.522 - 0.566)	0.083 (0.057 - 0.105)	0.682 (0.409 - 0.889)	0.080 (0.069 - 0.087)	0.318 (0.047 - 0.591)	0.695 (0.439 - 0.990)	0.043 (0.039 - 0.047)
**COST-SENSITIVE LR W/ ADASYN**	Test	0.535 (0.418 - 0.554)	0.408 (0.311 - 0.503)	0.669 (0.407 - 0.939)	0.089 (0.078 - 0.100)	0.330 (0.074 - 0.593)	0.759 (0.553 - 0.965)	0.048 (0.041 - 0.055)
	Train	0.559 (0.547 - 0.572)	0.088 (0.066 - 0.110)	0.675 (0.398 - 0.887)	0.088 (0.082 - 0.093)	0.325 (0.048 - 0.601)	0.739 (0.486 - 0.990)	0.047 (0.043 - 0.052)
**COST-SENSITIVE LR W/ SMOTEENN**	Test	0.536 (0.517 - 0.567)	0.467 (0.423 - 0.512)	0.782 (0.602 - 0.953)	0.087 (0.078 - 0.098)	0.218 (0.052 - 0.449)	0.884 (0.786 - 0.982)	0.046 (0.041 - 0.054)
	Train	0.544 (0.525 - 0.567)	0.081 (0.055 - 0.101)	0.742 (0.538 - 0.902)	0.084 (0.082 - 0.086)	0.258 (0.052 - 0.458)	0.783 (0.587 - 0.990)	0.045 (0.043 - 0.046)
**COST-SENSITIVE LR W/ RUS**	Test	0.510 (0.500 - 0.523)	0.521 (0.520 - 0.521)	0.939 (0.921 - 0.958)	0.080 (0.079 - 0.081)	0.061 (0.042 - 0.080)	1.000 (1.000 - 1.000)	0.042 (0.041 - 0.042)
	Train	0.534 (0.520 - 0.549)	0.079 (0.059 - 0.097)	0.957 (0.956 - 0.957)	0.082 (0.081 - 0.082)	0.043 (0.043 - 0.044)	0.996 (0.988 - 1.000)	0.043 (0.042 - 0.043)

LR: Logistic Regression, SMOTE: Synthetic Minority Over-sampling Technique, ADASYN: Adaptive Synthetic, RUS: Random under-Sampling, SMOTEENN: Synthetic Minority Over-sampling Technique Edited Nearest Neighbor, AUC-ROC: Area Under the Curve – Receiver Operative Curve, AUC-PR: Area Under the Curve – Precision-Recall Curve, w/: with, w/o: without, prob.cal.: probability calibration. Results obtained on test and training sets. For each metric mean value and its 95% confidence interval are reported.

TABLE S2 ENHANCED DECISION TREE PERFORMANCE

MODEL		ROC-AUC	PR-AUC	BRIER SCORE	F1 SCORE	ACCURACY	RECALL	PRECISION
**DT W/ SMOTE W/O PROB. CAL.**	Test	**0.541 (0.509 - 0.567)**	0.317 (0.123 - 0.425)	0.486 (0.246 - 0.642)	0.082 (0.066 - 0.101)	0.513 (0.341 - 0.651)	0.572 (0.179 - 0.780)	0.045 (0.033 - 0.052)
	Train	0.552 (0.517 - 0.587)	0.318 (0.312 - 0.330)	0.303 (0.283 - 0.318)	0.093 (0.087 - 0.100)	0.697 (0.682 - 0.711)	0.356 (0.333 - 0.378)	0.954 (0.050 - 0.061)
**DT W/ ADASYN W/O PROB. CAL.**	Test	0.516 (0.503 - 0.532)	0.324 (0.246 - 0.403)	0.550 (0.442 - 0.659)	0.080 (0.074 - 0.085)	0.450 (0.341 - 0.558)	0.590 (0.429 - 0.750)	0.042 (0.040 - 0.045)
	Train	0.524 (0.509 - 0.550)	0.262 (0.252 - 0.272)	0.531 (0.511 - 0.551)	0.092 (0.084 - 0.099)	0.669 (0.649 - 0.689)	0.655 (0.589 - 0.721)	0.047 (0.043 - 0.050)
**DT W/ SMOTEENN W/O PROB. CAL.**	Test	0.533 (0.517 - 0.549)	0.395 (0.320 - 0.470)	0.631 (0.453 - 0.810)	0.088 (0.083 - 0.094)	0.369 (0.190 - 0.547)	0.732 (0.571 - 0.893)	0.047 (0.043 - 0.051)
	Train	0.555 (0.520 - 0.590)	0.393 (0.387 - 0.399)	0.387 (0.339 - 0.435)	0.186 (0.170 - 0.201)	0.613 (0.565 - 0.661)	0.631 (0.489 - 0.773)	0.048 (0.040 - 0.056)
**DT W/ RUS W/O PROB. CAL.**	Test	0.511 (0.510 - 0.512)	0.325 (0.129 - 0.521)	0.560 (0.185 - 0.936)	0.077 (0.073 - 0.081)	0.440 (0.064 - 0.815)	0.590 (0.179 - 1.000)	0.044 (0.042 - 0.046)
	Train	0.535 (0.516 - 0.554)	0.228 (0.221 - 0.236)	0.436 (0.381 - 0.491)	0.093 (0.084 - 0.103)	0.564 (0.509 - 0.619)	0.506 (0.501 - 0.511)	0.052 (0.046 - 0.059)
**DT W/ SMOTE W/ PROB. CAL.**	Test	0.530 (0.512 - 0.545)	0.224 (0.162 - 0.280)	0.328 (0.261 - 0.401)	0.084 (0.074 - 0.090)	0.672 (0.589 - 0.723)	0.375 (0.267 - 0.496)	0.048 (0.042 - 0.051)
	Train	0.549 (0.520 - 0.578)	0.261 (0.146 - 0.376)	0.352 (0.268 - 0.452)	0.092 (0.061 - 0.109)	0.698 (0.588 - 0.792)	0.435 (0.255 - 0.598)	0.052 (0.036 - 0.061)
**DT W/ ADASYN W/ PROB. CAL.**	Test	0.530 (0.515 - 0.551)	0.245 (0.220 - 0.275)	0.369 (0.326 - 0.416)	0.085 (0.077 - 0.090)	0.631 (0.587 - 0.682)	0.420 (0.375 - 0.500)	0.047 (0.043 - 0.050)
	Train	0.559 (0.517 - 0.609)	0.191 (0.147 - 0.251)	0.351 (0.246 - 0.545)	0.090 (0.070 - 0.108)	0.649 (0.498 - 0.752)	0.500 (0.346 - 0.635)	0.052 (0.041 - 0.062)
**DT W/ SMOTEENN W/ PROB. CAL.**	Test	0.536 (0.509 - 0.565)	0.391 (0.283 - 0.490)	0.637 (0.436 - 0.859)	0.087 (0.080 - 0.094)	0.363 (0.124 - 0.646)	0.723 (0.500 - 0.961)	0.047 (0.042 - 0.051)
	Train	0.559 (0.518 - 0.601)	0.229 (0.045 - 0.428)	0.526 (0.371 - 0.723)	0.092 (0.069 - 0.113)	0.474 (0.277 - 0.671)	0.765 (0.526 - 0.970)	0.045 (0.032 - 0.056)
**DT W/ RUS W/ PROB. CAL.**	Test	0.508 (0.501 - 0.515)	0.244 (0.155 - 0.398)	0.482 (0.284 - 0.690)	0.065 (0.054 - 0.078)	0.518 (0.294 - 0.698)	0.428 (0.245 - 0.741)	0.036 (0.030 - 0.043)
	Train	0.517 (0.501 - 0.544)	0.067 (0.047 - 0.088)	0.470 (0.454 - 0.489)	0.081 (0.074 - 0.089)	0.530 (0.515 - 0.546)	0.475 (0.427 - 0.522)	0.045 (0.040 - 0.049)
**COST-SENSITIVE DT W/ SMOTE**	Test	0.535 (0.505 - 0.564)	0.395 (0.277 - 0.513)	0.645 (0.450 - 0.908)	0.087 (0.079 - 0.099)	0.355 (0.145 - 0.592)	0.732 (0.477 - 0.982)	0.047 (0.042 - 0.056)
	Train	0.557 (0.507 - 0.587)	0.235 (0.085 - 0.364)	0.545 (0.297 - 0.792)	0.095 (0.084 - 0.106)	0.455 (0.208 - 0.701)	0.625 (0.406 - 0.830)	0.053 (0.045 - 0.061)
**COST-SENSITIVE DT W/ ADASYN**	Test	0.524 (0.508 - 0.535)	0.449 (0.355 - 0.521)	0.775 (0.562 - 0.937)	0.083 (0.080 - 0.085)	0.225 (0.063 - 0.388)	0.848 (0.652 - 1.000)	0.044 (0.042 - 0.045)
	Train	0.529 (0.518 - 0.540)	0.382 (0.236 - 0.544)	0.676 (0.436 - 0.834)	0.084 (0.077 - 0.093)	0.324 (0.156 - 0.471)	0.703 (0.472 - 0.854)	0.046 (0.041 - 0.053)
**COST-SENSITIVE DT W/ SMOTEENN**	Test	0.510 (0.496 - 0.523)	0.512 (0.504 - 0.521)	0.923 (0.881 - 0.951)	0.080 (0.077 - 0.083)	0.077 (0.052 - 0.119)	0.982 (0.964 - 1.000)	0.042 (0.040 - 0.043)
	Train	0.525 (0.508 - 0.541)	0.420 (0.230 - 0.550)	0.694 (0.431 - 0.838)	0.085 (0.079 - 0.091)	0.306 (0.162 - 0.572)	0.839 (0.771 - 0.907)	0.046 (0.041 - 0.049)
**COST-SENSITIVE DT W/ RUS**	Test	0.507 (0.500 - 0.515)	0.504 (0.469 - 0.521)	0.913 (0.847 - 0.959)	0.079 (0.078 - 0.080)	0.087 (0.041 - 0.154)	0.964 (0.893 - 1.000)	0.042 (0.041 - 0.042)
	Train	0.513 (0.505 - 0.521)	0.384 (0.236 - 0.502)	0.846 (0.719 - 0.919)	0.082 (0.080 - 0.084)	0.154 (0.078 - 0.281)	0.878 (0.764 - 0.959)	0.043 (0.042 - 0.044)

DT: Decision Tree, SMOTE: Synthetic Minority Over-sampling Technique, ADASYN: Adaptive Synthetic, RUS: Random under-Sampling, SMOTEENN: Synthetic Minority Over-sampling Technique Edited Nearest Neighbor, AUC-ROC: Area Under the Curve – Receiver Operative Curve, AUC-PR: Area Under the Curve – Precision-Recall Curve, w/: with, w/o: without, prob.cal.: probability calibration. Results obtained on test and training sets. For each metric mean value and its 95% confidence interval are reported.

TABLE S3
ENHANCED RANDOM FOREST PERFORMANCE

MODEL		AUC-ROC	AUC-PR	BRIER SCORE	F1 SCORE	ACCURACY	RECALL	PRECISION
**RF W/ SMOTE W/O PROB. CAL.**	Test	0.545<br>(0.521 - 0.570)	0.386<br>(0.368 - 0.405)	0.610<br>(0.596 - 0.623)	0.088<br>(0.082 - 0.094)	0.391<br>(0.377 - 0.404)	0.715<br>(0.679 - 0.750)	0.046<br>(0.043 - 0.050)
	Train	0.565<br>(0.555 - 0.576)	0.372<br>(0.362 - 0.381)	0.596<br>(0.521 - 0.672)	0.093<br>(0.083 - 0.103)	0.604<br>(0.528 - 0.679)	0.703<br>(0.682 - 0.723)	0.047<br>(0.035 - 0.058)
**RF W/ ADASYN W/O PROB. CAL.**	Test	0.516<br>(0.508 - 0.523)	0.333<br>(0.332 - 0.334)	0.569<br>(0.536 - 0.601)	0.081<br>(0.076 - 0.085)	0.431<br>(0.399 - 0.464)	0.607<br>(0.607 - 0.607)	0.043<br>(0.041 - 0.046)
	Train	0.549<br>(0.544 - 0.554)	0.290<br>(0.289 - 0.290)	0.437<br>(0.397 - 0.478)	0.076<br>(0.071 - 0.080)	0.563<br>(0.522 - 0.603)	0.559<br>(0.496 - 0.622)	0.056<br>(0.054 - 0.059)
**RF W/ SMOTEENN W/O PROB. CAL.**	Test	0.532<br>(0.505 - 0.560)	0.461<br>(0.416 - 0.506)	0.816<br>(0.811 - 0.820)	0.081<br>(0.073 - 0.089)	0.184<br>(0.180 - 0.189)	0.875<br>(0.786 - 0.964)	0.042<br>(0.038 - 0.047)
	Train	0.588<br>(0.585 - 0.591)	0.375<br>(0.342 - 0.409)	0.696<br>(0.644 - 0.748)	0.076<br>(0.076 - 0.077)	0.304<br>(0.252 - 0.356)	0.767<br>(0.713 - 0.821)	0.041<br>(0.040 - 0.041)
**RF W/ RUS W/O PROB. CAL.**	Test	**0.642**<br>**(0.504 - 0.781)**	0.337<br>(0.121 - 0.553)	0.308<br>(0.274 - 0.343)	0.122<br>(0.051 - 0.193)	0.692<br>(0.657 - 0.726)	0.590<br>(0.179 - 1.000)	0.068<br>(0.029 - 0.107)
	Train	0.666<br>(0.555 - 0.777)	0.174<br>(0.158 - 0.190)	0.302<br>(0.270 - 0.335)	0.088<br>(0.087 - 0.090)	0.598<br>(0.565 - 0.630)	0.562<br>(0.527 - 0.596)	0.049<br>(0.047 - 0.051)
**RF W/ SMOTE W/ PROB. CAL.**	Test	0.532<br>(0.524 - 0.540)	0.309<br>(0.232 - 0.386)	0.487<br>(0.355 - 0.619)	0.084<br>(0.083 - 0.086)	0.513<br>(0.381 - 0.645)	0.553<br>(0.393 - 0.714)	0.046<br>(0.046 - 0.046)
	Train	0.594<br>(0.587 - 0.600)	0.249<br>(0.240 - 0.257)	0.387<br>(0.387 - 0.388)	0.081<br>(0.077 - 0.085)	0.613<br>(0.612 - 0.613)	0.406<br>(0.388 - 0.424)	0.045<br>(0.043 - 0.048)
**RF W/ ADASYN W/ PROB. CAL.**	Test	0.535<br>(0.500 - 0.571)	0.394<br>(0.366 - 0.422)	0.645<br>(0.625 - 0.664)	0.085<br>(0.077 - 0.093)	0.356<br>(0.336 - 0.375)	0.733<br>(0.679 - 0.786)	0.045<br>(0.041 - 0.050)
	Train	0.543<br>(0.502 - 0.564)	0.252<br>(0.235 - 0.269)	0.342<br>(0.203 - 0.480)	0.079<br>(0.057 - 0.101)	0.658<br>(0.520 - 0.797)	0.515<br>(0.468 - 0.562)	0.047<br>(0.031 - 0.063)
**RF W/ SMOTEENN W/ PROB. CAL.**	Test	0.567<br>(0.505 - 0.628)	0.379<br>(0.264 - 0.494)	0.554<br>(0.458 - 0.649)	0.090<br>(0.076 - 0.105)	0.447<br>(0.351 - 0.542)	0.697<br>(0.464 - 0.929)	0.049<br>(0.042 - 0.056)
	Train	0.582<br>(0.580 - 0.584)	0.285<br>(0.274 - 0.296)	0.592<br>(0.554 - 0.630)	0.076<br>(0.073 - 0.079)	0.408<br>(0.370 - 0.446)	0.563<br>(0.562 - 0.564)	0.041<br>(0.039 - 0.043)
**RF W/ RUS W/ PROB. CAL.**	Test	0.606<br>(0.597 - 0.615)	0.467<br>(0.441 - 0.493)	0.640<br>(0.609 - 0.672)	0.101<br>(0.099 - 0.102)	0.360<br>(0.328 - 0.391)	0.875<br>(0.821 - 0.929)	0.053<br>(0.053 - 0.054)
	Train	0.593<br>(0.588 - 0.608)	0.349<br>(0.349 - 0.350)	0.505<br>(0.486 - 0.525)	0.088<br>(0.083 - 0.093)	0.495<br>(0.475 - 0.514)	0.760<br>(0.700 - 0.821)	0.048<br>(0.045 - 0.050)
**COST-SENSITIVE RF W/ SMOTE**	Test	0.533<br>(0.516 - 0.550)	0.455<br>(0.405 - 0.505)	0.726<br>(0.596 - 0.857)	0.089<br>(0.084 - 0.094)	0.274<br>(0.143 - 0.404)	0.857<br>(0.750 - 0.964)	0.047<br>(0.044 - 0.050)
	Train	0.558<br>(0.545 - 0.572)	0.464<br>(0.281 - 0.646)	0.660<br>(0.472 - 0.849)	0.071<br>(0.063 - 0.079)	0.340<br>(0.151 - 0.528)	0.813<br>(0.682 - 0.945)	0.038<br>(0.035 - 0.041)
**COST-SENSITIVE RF W/ ADASYN**	Test	0.534<br>(0.533 - 0.536)	0.420<br>(0.334 - 0.505)	0.696<br>(0.536 - 0.857)	0.085<br>(0.084 - 0.085)	0.303<br>(0.143 - 0.464)	0.785<br>(0.607 - 0.964)	0.045<br>(0.044 - 0.046)
	Train	0.569<br>(0.554 - 0.583)	0.253<br>(0.189 - 0.317)	0.647<br>(0.478 - 0.817)	0.075<br>(0.070 - 0.080)	0.353<br>(0.183 - 0.522)	0.626<br>(0.422 - 0.829)	0.040<br>(0.039 - 0.042)
**COST-SENSITIVE RF W/ SMOTEENN**	Test	0.515<br>(0.500 - 0.530)	0.461<br>(0.416 - 0.506)	0.816<br>(0.811 - 0.820)	0.081<br>(0.073 - 0.089)	0.184<br>(0.180 - 0.189)	0.875<br>(0.786 - 0.964)	0.042<br>(0.038 - 0.047)
	Train	0.588<br>(0.585 - 0.591)	0.375<br>(0.342 - 0.409)	0.696<br>(0.644 - 0.748)	0.076<br>(0.076 - 0.077)	0.304<br>(0.252 - 0.356)	0.767<br>(0.713 - 0.821)	0.041<br>(0.040 - 0.041)
**COST-SENSITIVE RF W/ RUS**	Test	0.506<br>(0.501 - 0.512)	0.512<br>(0.504 - 0.521)	0.931<br>(0.904 - 0.958)	0.080<br>(0.079 - 0.080)	0.069<br>(0.042 - 0.096)	0.982<br>(0.964 - 1.000)	0.042<br>(0.041 - 0.042)
	Train	0.515<br>(0.511 - 0.519)	0.536<br>(0.474 - 0.598)	0.923<br>(0.916 - 0.930)	0.082<br>(0.082 - 0.083)	0.077<br>(0.070 - 0.084)	0.985<br>(0.969 - 1.000)	0.043<br>(0.043 - 0.043)

RF: Random Forest, SMOTE: Synthetic Minority Over-sampling Technique, ADASYN: Adaptive Synthetic, RUS: Random under-Sampling, SMOTEENN: Synthetic Minority Over-sampling Technique Edited Nearest Neighbor, AUC-ROC: Area Under the Curve – Receiver Operative Curve, AUC-PR: Area Under the Curve – Precision-Recall Curve, w/: with, w/o: without, prob.cal.: probability calibration. Results obtained on test and training sets. For each metric mean value and its 95% confidence interval are reported.

TABLE S4 ENHANCED ADAPTIVE BOOSTING PERFORMANCE

MODEL		AUC-ROC	AUC-PR	BRIER SCORE	F1 SCORE	ACCURACY	RECALL	PRECISION
**ADABOOST W/ SMOTE W/O PROB. CAL.**	Test	0.540 (0.515 - 0.590)	0.375 (0.333 - 0.419)	0.598 (0.504 - 0.731)	0.087 (0.080 - 0.101)	0.402 (0.269 - 0.508)	0.691 (0.607 - 0.769)	0.047 (0.043 - 0.055)
	Train	0.583 (0.567 - 0.595)	0.324 (0.286 - 0.364)	0.440 (0.380 - 0.498)	0.098 (0.086 - 0.114)	0.560 (0.520 - 0.620)	0.575 (0.505 - 0.658)	0.054 (0.042 - 0.063)
**ADABOOST W/ ADASYN W/O PROB. CAL.**	Test	0.541 (0.515 - 0.590)	0.375 (0.333 - 0.419)	0.596 (0.504 - 0.726)	0.087 (0.080 - 0.101)	0.404 (0.274 - 0.508)	0.691 (0.607 - 0.769)	0.047 (0.043 - 0.055)
	Train	0.588 (0.581 - 0.594)	0.316 (0.281 - 0.335)	0.407 (0.380 - 0.459)	0.102 (0.093 - 0.114)	0.593 (0.566 - 0.619)	0.555 (0.487 - 0.596)	0.057 (0.050 - 0.064)
**ADABOOST W/ SMOTEENN W/O PROB. CAL.**	Test	0.518 (0.513 - 0.525)	0.322 (0.265 - 0.351)	0.541 (0.456 - 0.609)	0.081 (0.079 - 0.082)	0.459 (0.391 - 0.568)	0.583 (0.464 - 0.643)	0.043 (0.043 - 0.044)
	Train	0.580 (0.537 - 0.614)	0.230 (0.150 - 0.324)	0.469 (0.316 - 0.585)	0.100 (0.090 - 0.113)	0.531 (0.435 - 0.684)	0.588 (0.483 - 0.690)	0.055 (0.047 - 0.064)
**ADABOOST W/ RUS W/O PROB. CAL.**	Test	0.539 (0.441 - 0.614)	0.274 (0.137 - 0.386)	0.405 (0.257 - 0.565)	0.091 (0.061 - 0.129)	0.595 (0.394 - 0.743)	0.476 (0.214 - 0.714)	0.051 (0.027 - 0.069)
	Train	0.557 (0.540 - 0.577)	0.158 (0.061 - 0.286)	0.409 (0.374 - 0.452)	0.096 (0.093 - 0.100)	0.591 (0.548 - 0.619)	0.514 (0.483 - 0.537)	0.055 (0.052 - 0.058)
**ADABOOST W/ SMOTE W/ PROB. CAL.**	Test	0.535 (0.500 - 0.590)	0.352 (0.264 - 0.419)	0.563 (0.466 - 0.731)	0.086 (0.077 - 0.101)	0.437 (0.269 - 0.525)	0.643 (0.464 - 0.769)	0.046 (0.041 - 0.055)
	Train	0.598 (0.582 - 0.610)	0.257 (0.185 - 0.304)	0.411 (0.382 - 0.441)	0.100 (0.086 - 0.118)	0.589 (0.569 - 0.618)	0.548 (0.473 - 0.594)	0.055 (0.044 - 0.066)
**ADABOOST W/ ADASYN W/ PROB. CAL.**	Test	**0.543 (0.520 - 0.582)**	0.364 (0.300 - 0.419)	0.571 (0.481 - 0.726)	0.087 (0.081 - 0.098)	0.429 (0.274 - 0.510)	0.667 (0.536 - 0.769)	0.047 (0.044 - 0.053)
	Train	0.591 (0.574 - 0.599)	0.154 (0.141 - 0.171)	0.433 (0.387 - 0.469)	0.099 (0.096 - 0.102)	0.567 (0.537 - 0.613)	0.553 (0.471 - 0.644)	0.055 (0.053 - 0.057)
**ADABOOST W/ SMOTEENN W/ PROB. CAL.**	Test	0.519 (0.504 - 0.540)	0.357 (0.346 - 0.373)	0.606 (0.506 - 0.745)	0.083 (0.069 - 0.101)	0.394 (0.255 - 0.508)	0.655 (0.643 - 0.679)	0.045 (0.038 - 0.055)
	Train	0.589 (0.570 - 0.604)	0.221 (0.192 - 0.248)	0.549 (0.491 - 0.616)	0.099 (0.095 - 0.106)	0.451 (0.404 - 0.509)	0.693 (0.642 - 0.782)	0.054 (0.050 - 0.058)
**ADABOOST W/ RUS W/ PROB. CAL.**	Test	0.528 (0.501 - 0.554)	0.274 (0.137 - 0.386)	0.405 (0.257 - 0.565)	0.091 (0.061 - 0.129)	0.595 (0.394 - 0.743)	0.476 (0.214 - 0.714)	0.051 (0.027 - 0.069)
	Train	0.557 (0.540 - 0.577)	0.158 (0.061 - 0.286)	0.409 (0.374 - 0.452)	0.096 (0.093 - 0.100)	0.591 (0.548 - 0.619)	0.514 (0.483 - 0.537)	0.055 (0.052 - 0.058)

AdaBoost: Adaptive Boosting, SMOTE: Synthetic Minority Over-sampling Technique, ADASYN: Adaptive Synthetic, RUS: Random under-Sampling, SMOTEENN: Synthetic Minority Over-sampling Technique Edited Nearest Neighbor, AUC-ROC: Area Under the Curve – Receiver Operative Curve, AUC-PR: Area Under the Curve – Precision-Recall Curve, w/: with, w/o: without, prob.cal.: probability calibration. Results obtained on test and training sets. For each metric mean value and its 95% confidence interval are reported.

TABLE S5 ENHANCED LOGISTIC REGRESSION WITH DATA AUGMENTATION PERFORMANCE

MODEL		AUC-ROC	AUC-PR	BRIER SCORE	F1 SCORE	ACCURACY	RECALL	PRECISION
**LR W/ SMOTE W/O PROB. CAL.**	Test	**0.539 (0.510 - 0.559)**	0.262 (0.247 - 0.288)	0.382 (0.361 - 0.400)	0.089 (0.078 - 0.099)	0.618 (0.585 - 0.636)	0.453 (0.429 - 0.500)	0.049 (0.045 - 0.056)
	Train	0.568 (0.515 - 0.617)	0.294 (0.269 - 0.321)	0.379 (0.348 - 0.410)	0.095 (0.079 - 0.121)	0.621 (0.560 - 0.653)	0.452 (0.400 - 0.504)	0.053 (0.044 - 0.068)
**LR W/ ADASYN W/O PROB. CAL.**	Test	**0.539 (0.523 - 0.555)**	0.273 (0.260 - 0.288)	0.403 (0.368 - 0.432)	0.089 (0.082 - 0.097)	0.597 (0.544 - 0.627)	0.476 (0.429 - 0.500)	0.049 (0.046 - 0.055)
	Train	0.563 (0.516 - 0.617)	0.287 (0.267 - 0.310)	0.396 (0.370 - 0.422)	0.096 (0.077 - 0.126)	0.604 (0.562 - 0.640)	0.475 (0.378 - 0.547)	0.054 (0.043 - 0.070)
**LR W/ SMOTEENN W/O PROB. CAL.**	Test	0.515 (0.505 - 0.520)	0.322 (0.284 - 0.355)	0.547 (0.421 - 0.706)	0.081 (0.077 - 0.087)	0.453 (0.294 - 0.548)	0.583 (0.500 - 0.714)	0.043 (0.040 - 0.048)
	Train	0.556 (0.514 - 0.591)	0.287 (0.262 - 0.317)	0.505 (0.454 - 0.556)	0.093 (0.083 - 0.109)	0.495 (0.399 - 0.552)	0.585 (0.547 - 0.613)	0.051 (0.045 - 0.059)
**LR W/ RUS W/O PROB. CAL.**	Test	0.538 (0.501 - 0.550)	0.190 (0.152 - 0.241)	0.437 (0.416 - 0.457)	0.088 (0.066 - 0.104)	0.563 (0.543 - 0.576)	0.512 (0.393 - 0.607)	0.048 (0.038 - 0.058)
	Train	0.594 (0.538 - 0.625)	0.207 (0.200 - 0.216)	0.427 (0.389 - 0.462)	0.102 (0.090 - 0.114)	0.573 (0.508 - 0.607)	0.560 (0.505 - 0.592)	0.056 (0.049 - 0.064)
**LR W/ SMOTE W/ PROB. CAL.**	Test	0.530 (0.523 - 0.538)	0.255 (0.231 - 0.284)	0.389 (0.369 - 0.408)	0.085 (0.081 - 0.088)	0.611 (0.573 - 0.631)	0.441 (0.393 - 0.500)	0.047 (0.044 - 0.048)
	Train	0.559 (0.526 - 0.592)	0.200 (0.171 - 0.233)	0.378 (0.346 - 0.409)	0.096 (0.080 - 0.122)	0.622 (0.563 - 0.658)	0.454 (0.400 - 0.503)	0.054 (0.044 - 0.069)
**LR W/ ADASYN W/ PROB. CAL.**	Test	0.524 (0.506 - 0.542)	0.229 (0.217 - 0.246)	0.412 (0.368 - 0.440)	0.073 (0.066 - 0.079)	0.588 (0.548 - 0.616)	0.393 (0.357 - 0.429)	0.040 (0.036 - 0.045)
	Train	0.566 (0.518 - 0.614)	0.201 (0.187 - 0.217)	0.379 (0.369 - 0.395)	0.103 (0.095 - 0.115)	0.621 (0.612 - 0.634)	0.459 (0.333 - 0.532)	0.060 (0.056 - 0.064)
**LR W/ SMOTEENN W/ PROB. CAL.**	Test	0.529 (0.509 - 0.545)	0.317 (0.299 - 0.335)	0.509 (0.425 - 0.587)	0.085 (0.079 - 0.091)	0.491 (0.413 - 0.545)	0.571 (0.536 - 0.643)	0.046 (0.043 - 0.051)
	Train	0.565 (0.517 - 0.612)	0.090 (0.068 - 0.118)	0.518 (0.455 - 0.581)	0.093 (0.082 - 0.109)	0.482 (0.364 - 0.552)	0.592 (0.562 - 0.615)	0.051 (0.044 - 0.059)
**LR W/ RUS W/ PROB. CAL.**	Test	0.507 (0.504 - 0.511)	0.263 (0.210 - 0.305)	0.474 (0.434 - 0.524)	0.073 (0.066 - 0.087)	0.526 (0.476 - 0.595)	0.464 (0.357 - 0.607)	0.040 (0.036 - 0.045)
	Train	0.528 (0.507 - 0.548)	0.258 (0.246 - 0.275)	0.412 (0.361 - 0.462)	0.088 (0.077 - 0.101)	0.588 (0.508 - 0.660)	0.458 (0.292 - 0.551)	0.049 (0.043 - 0.057)
**COST-SENSITIVE LR W/ SMOTE**	Test	0.535 (0.502 - 0.558)	0.353 (0.263 - 0.521)	0.563 (0.365 - 0.760)	0.089 (0.079 - 0.098)	0.437 (0.044 - 0.634)	0.643 (0.429 - 1.000)	0.048 (0.043 - 0.056)
	Train	0.568 (0.507 - 0.629)	0.286 (0.263 - 0.313)	0.580 (0.380 - 0.950)	0.091 (0.076 - 0.117)	0.420 (0.220 - 0.650)	0.641 (0.400 - 0.986)	0.050 (0.041 - 0.066)
**COST-SENSITIVE LR W/ ADASYN**	Test	0.514 (0.506 - 0.522)	0.503 (0.468 - 0.521)	0.939 (0.901 - 0.959)	0.078 (0.076 - 0.079)	0.061 (0.041 - 0.081)	0.964 (0.893 - 1.000)	0.040 (0.039 - 0.041)
	Train	0.562 (0.519 - 0.601)	0.376 (0.363 - 0.392)	0.778 (0.607 - 0.951)	0.079 (0.074 - 0.084)	0.222 (0.050 - 0.562)	0.788 (0.378 - 0.995)	0.042 (0.040 - 0.044)
**COST-SENSITIVE LR W/ SMOTEENN**	Test	0.528 (0.502 - 0.554)	0.460 (0.339 - 0.521)	0.786 (0.445 - 0.956)	0.086 (0.079 - 0.097)	0.214 (0.044 - 0.385)	0.869 (0.607 - 1.000)	0.045 (0.041 - 0.054)
	Train	0.565 (0.518 - 0.613)	0.384 (0.362 - 0.408)	0.668 (0.504 - 0.947)	0.090 (0.079 - 0.111)	0.332 (0.169 - 0.544)	0.732 (0.578 - 0.957)	0.048 (0.042 - 0.061)
**COST-SENSITIVE LR W/ RUS**	Test	0.531 (0.511 - 0.550)	0.423 (0.325 - 0.520)	0.792 (0.457 - 0.959)	0.074 (0.066 - 0.079)	0.208 (0.041 - 0.543)	0.798 (0.393 - 1.000)	0.039 (0.037 - 0.041)
	Train	0.583 (0.538 - 0.622)	0.391 (0.380 - 0.401)	0.801 (0.646 - 0.956)	0.082 (0.080 - 0.085)	0.199 (0.043 - 0.508)	0.835 (0.505 - 1.000)	0.044 (0.043 - 0.045)

LR: Logistic Regression, SMOTE: Synthetic Minority Over-sampling Technique, ADASYN: Adaptive Synthetic, RUS: Random under-Sampling, SMOTEENN: Synthetic Minority Over-sampling Technique Edited Nearest Neighbor, AUC-ROC: Area Under the Curve – Receiver Operative Curve, AUC-PR: Area Under the Curve – Precision-Recall Curve, w/: with, w/o: without, prob.cal.: probability calibration. Results obtained on test and training sets. For each metric mean value and its 95% confidence interval are reported.

TABLE S6 ENHANCED DECISION TREE WITH DATA AUGMENTATION PERFORMANCE

MODEL		AUC-ROC	AUC-PR	BRIER SCORE	F1 SCORE	ACCURACY	RECALL	PRECISION
**DT W/ SMOTE W/O PROB. CAL.**	Test	0.514 (0.503 - 0.525)	0.183 (0.104 - 0.283)	0.287 (0.107 - 0.453)	0.066 (0.027 - 0.088)	0.712 (0.597 - 0.893)	0.298 (0.036 - 0.500)	0.039 (0.025 - 0.048)
	Train	0.522 (0.506 - 0.537)	0.171 (0.159 - 0.180)	0.293 (0.284 - 0.306)	0.079 (0.059 - 0.107)	0.707 (0.699 - 0.720)	0.289 (0.176 - 0.429)	0.046 (0.034 - 0.066)
**DT W/ ADASYN W/O PROB. CAL.**	Test	0.519 (0.508 - 0.539)	0.317 (0.191 - 0.521)	0.528 (0.242 - 0.937)	0.080 (0.068 - 0.092)	0.472 (0.240 - 0.758)	0.571 (0.214 - 1.000)	0.044 (0.040 - 0.047)
	Train	0.536 (0.512 - 0.560)	0.298 (0.251 - 0.360)	0.329 (0.283 - 0.375)	0.081 (0.070 - 0.102)	0.671 (0.581 - 0.719)	0.341 (0.222 - 0.446)	0.047 (0.041 - 0.056)
**DT W/ SMOTEENN W/O PROB. CAL.**	Test	0.515 (0.502 - 0.527)	0.447 (0.349 - 0.521)	0.799 (0.701 - 0.942)	0.079 (0.075 - 0.083)	0.201 (0.102 - 0.354)	0.845 (0.643 - 1.000)	0.041 (0.040 - 0.043)
	Train	0.554 (0.510 - 0.598)	0.380 (0.366 - 0.392)	0.361 (0.346 - 0.386)	0.096 (0.079 - 0.116)	0.639 (0.623 - 0.661)	0.442 (0.289 - 0.592)	0.054 (0.045 - 0.067)
**DT W/ RUS W/O PROB. CAL.**	Test	0.510 (0.500 - 0.518)	0.253 (0.109 - 0.520)	0.109 (0.057 - 0.195)	0.053 (0.000 - 0.085)	0.891 (0.805 - 0.959)	0.095 (0.000 - 0.179)	0.039 (0.014 - 0.075)
	Train	0.519 (0.512 - 0.529)	0.245 (0.201 - 0.267)	0.415 (0.310 - 0.487)	0.081 (0.080 - 0.081)	0.585 (0.473 - 0.657)	0.432 (0.324 - 0.511)	0.046 (0.045 - 0.049)
**DT W/ SMOTE W/ PROB. CAL.**	Test	0.519 (0.508 - 0.529)	0.334 (0.208 - 0.520)	0.256 (0.108 - 0.485)	0.054 (0.020 - 0.089)	0.744 (0.596 - 0.959)	0.274 (0.100 - 0.507)	0.030 (0.013 - 0.050)
	Train	0.575 (0.515 - 0.635)	0.298 (0.243 - 0.406)	0.245 (0.230 - 0.271)	0.063 (0.048 - 0.088)	0.755 (0.741 - 0.773)	0.214 (0.133 - 0.257)	0.055 (0.036 - 0.083)
**DT W/ ADASYN W/ PROB. CAL.**	Test	0.514 (0.509 - 0.519)	0.237 (0.212 - 0.284)	0.385 (0.294 - 0.431)	0.080 (0.064 - 0.089)	0.615 (0.567 - 0.661)	0.405 (0.357 - 0.500)	0.045 (0.037 - 0.052)
	Train	0.522 (0.508 - 0.536)	0.206 (0.148 - 0.285)	0.366 (0.232 - 0.500)	0.069 (0.054 - 0.092)	0.634 (0.369 - 0.770)	0.345 (0.109 - 0.550)	0.040 (0.034 - 0.049)
**DT W/ SMOTEENN W/ PROB. CAL.**	Test	**0.530 (0.509 - 0.560)**	0.448 (0.371 - 0.503)	0.759 (0.630 - 0.936)	0.085 (0.078 - 0.092)	0.241 (0.112 - 0.452)	0.845 (0.679 - 0.964)	0.044 (0.040 - 0.047)
	Train	0.551 (0.539 - 0.564)	0.362 (0.344 - 0.386)	0.350 (0.280 - 0.403)	0.087 (0.066 - 0.108)	0.650 (0.560 - 0.703)	0.692 (0.500 - 0.820)	0.049 (0.037 - 0.063)
**DT W/ RUS W/ PROB. CAL.**	Test	0.515 (0.508 - 0.521)	0.355 (0.282 - 0.455)	0.636 (0.459 - 0.726)	0.078 (0.065 - 0.088)	0.364 (0.269 - 0.455)	0.655 (0.500 - 0.857)	0.041 (0.036 - 0.045)
	Train	0.528 (0.519 - 0.536)	0.260 (0.252 - 0.270)	0.479 (0.462 - 0.508)	0.080 (0.069 - 0.087)	0.521 (0.504 - 0.544)	0.478 (0.397 - 0.531)	0.044 (0.036 - 0.048)
**COST-SENSITIVE DT W/ SMOTE**	Test	0.523 (0.509 - 0.534)	0.510 (0.488 - 0.521)	0.893 (0.828 - 0.936)	0.082 (0.081 - 0.084)	0.107 (0.058 - 0.172)	0.976 (0.929 - 1.000)	0.043 (0.042 - 0.044)
	Train	0.543 (0.515 - 0.571)	0.419 (0.370 - 0.502)	0.868 (0.828 - 0.920)	0.083 (0.080 - 0.085)	0.132 (0.099 - 0.179)	0.898 (0.864 - 0.944)	0.044 (0.043 - 0.044)
**COST-SENSITIVE DT W/ ADASYN**	Test	0.521 (0.505 - 0.552)	0.476 (0.443 - 0.521)	0.831 (0.662 - 0.927)	0.082 (0.079 - 0.089)	0.169 (0.051 - 0.338)	0.905 (0.786 - 1.000)	0.043 (0.041 - 0.046)
	Train	0.595 (0.560 - 0.630)	0.333 (0.181 - 0.516)	0.723 (0.556 - 0.919)	0.079 (0.071 - 0.087)	0.277 (0.111 - 0.580)	0.744 (0.354 - 0.986)	0.042 (0.039 - 0.046)
**COST-SENSITIVE DT W/ SMOTEENN**	Test	0.529 (0.505 - 0.576)	0.454 (0.399 - 0.521)	0.771 (0.486 - 0.927)	0.085 (0.079 - 0.098)	0.228 (0.051 - 0.514)	0.857 (0.643 - 1.000)	0.045 (0.041 - 0.051)
	Train	0.570 (0.549 - 0.591)	0.398 (0.274 - 0.529)	0.745 (0.623 - 0.885)	0.081 (0.077 - 0.085)	0.255 (0.133 - 0.480)	0.760 (0.486 - 0.912)	0.043 (0.041 - 0.045)
**COST-SENSITIVE DT W/ RUS**	Test	0.523 (0.509 - 0.536)	0.378 (0.304 - 0.521)	0.723 (0.543 - 0.946)	0.077 (0.054 - 0.097)	0.277 (0.097 - 0.594)	0.702 (0.536 - 1.000)	0.041 (0.030 - 0.050)
	Train	0.535 (0.515 - 0.555)	0.281 (0.141 - 0.454)	0.711 (0.403 - 0.871)	0.087 (0.080 - 0.095)	0.289 (0.117 - 0.449)	0.759 (0.507 - 0.909)	0.047 (0.042 - 0.055)

DT: Decision Tree, SMOTE: Synthetic Minority Over-sampling Technique, ADASYN: Adaptive Synthetic, RUS: Random under-Sampling, SMOTEENN: Synthetic Minority Over-sampling Technique Edited Nearest Neighbor, AUC-ROC: Area Under the Curve – Receiver Operative Curve, AUC-PR: Area Under the Curve – Precision-Recall Curve, w/: with, w/o: without, prob.cal.: probability calibration. Results obtained on test and training sets. For each metric mean value and its 95% confidence interval are reported.

Table S7 Enhanced Random Forest with Data Augmentation Performance

MODEL		AUC-ROC	AUC-PR	BRIER SCORE	F1 SCORE	ACCURACY	RECALL	PRECISION
**RF W/ SMOTE W/O PROB. CAL.**	Test	0.530	0.341	0.551	0.086	0.450	0.619	0.047
		(0.511 - 0.554)	(0.237 - 0.408)	(0.411 - 0.717)	(0.078 - 0.097)	(0.283 - 0.702)	(0.393 - 0.821)	(0.040 - 0.052)
	Train	0.572	0.296	0.392	0.081	0.608	0.419	0.050
		(0.556 - 0.588)	(0.268 - 0.337)	(0.325 - 0.460)	(0.068 - 0.101)	(0.482 - 0.685)	(0.244 - 0.563)	(0.042 - 0.059)
**RF W/ ADASYN W/O PROB. CAL.**	Test	0.527	0.374	0.623	0.084	0.378	0.691	0.045
		(0.505 - 0.559)	(0.247 - 0.448)	(0.419 - 0.871)	(0.077 - 0.096)	(0.129 - 0.528)	(0.429 - 0.893)	(0.041 - 0.048)
	Train	0.550	0.363	0.355	0.093	0.645	0.404	0.053
		(0.506 - 0.595)	(0.345 - 0.387)	(0.312 - 0.377)	(0.074 - 0.114)	(0.621 - 0.667)	(0.289 - 0.467)	(0.042 - 0.067)
**RF W/ SMOTEENN W/O PROB. CAL.**	Test	0.511	0.330	0.655	0.068	0.345	0.607	0.037
		(0.502 - 0.520)	(0.157 - 0.416)	(0.320 - 0.829)	(0.062 - 0.074)	(0.156 - 0.520)	(0.250 - 0.786)	(0.034 - 0.039)
	Train	0.525	0.221	0.639	0.083	0.361	0.666	0.044
		(0.510 - 0.540)	(0.174 - 0.273)	(0.603 - 0.681)	(0.078 - 0.089)	(0.325 - 0.427)	(0.612 - 0.710)	(0.041 - 0.047)
**RF W/ RUS W/O PROB. CAL.**	Test	0.512	0.199	0.381	0.065	0.619	0.333	0.037
		(0.509 - 0.515)	(0.108 - 0.278)	(0.256 - 0.557)	(0.061 - 0.068)	(0.493 - 0.819)	(0.143 - 0.500)	(0.037 - 0.038)
	Train	0.564	0.168	0.547	0.091	0.453	0.624	0.049
		(0.543 - 0.596)	(0.162 - 0.176)	(0.493 - 0.593)	(0.087 - 0.099)	(0.371 - 0.498)	(0.519 - 0.704)	(0.047 - 0.054)
**RF W/ SMOTE W/ PROB. CAL.**	Test	**0.535**	0.280	0.402	0.090	0.598	0.488	0.050
		**(0.513 - 0.557)**	(0.176 - 0.336)	(0.328 - 0.468)	(0.069 - 0.123)	(0.475 - 0.663)	(0.286 - 0.607)	(0.038 - 0.065)
	Train	0.566	0.267	0.344	0.081	0.656	0.378	0.047
		(0.534 - 0.598)	(0.249 - 0.289)	(0.252 - 0.445)	(0.067 - 0.102)	(0.565 - 0.829)	(0.133 - 0.513)	(0.040 - 0.056)
**RF W/ ADASYN W/ PROB. CAL.**	Test	0.511	0.191	0.477	0.077	0.522	0.500	0.042
		(0.509 - 0.513)	(0.156 - 0.231)	(0.335 - 0.652)	(0.060 - 0.095)	(0.348 - 0.628)	(0.250 - 0.679)	(0.032 - 0.049)
	Train	0.518	0.176	0.372	0.073	0.628	0.356	0.041
		(0.509 - 0.527)	(0.156 - 0.196)	(0.297 - 0.447)	(0.057 - 0.089)	(0.551 - 0.776)	(0.109 - 0.499)	(0.034 - 0.049)
**RF W/ SMOTEENN W/ PROB. CAL.**	Test	0.518	0.385	0.661	0.081	0.339	0.714	0.043
		(0.502 - 0.537)	(0.366 - 0.403)	(0.638 - 0.687)	(0.076 - 0.085)	(0.323 - 0.362)	(0.679 - 0.750)	(0.039 - 0.045)
	Train	0.536	0.277	0.459	0.081	0.541	0.509	0.045
		(0.525 - 0.547)	(0.246 - 0.329)	(0.365 - 0.557)	(0.062 - 0.101)	(0.447 - 0.725)	(0.156 - 0.693)	(0.035 - 0.055)
**RF W/ RUS W/ PROB. CAL.**	Test	0.519	0.272	0.567	0.064	0.433	0.488	0.034
		(0.502 - 0.536)	(0.194 - 0.384)	(0.504 - 0.621)	(0.039 - 0.081)	(0.335 - 0.496)	(0.250 - 0.714)	(0.024 - 0.041)
	Train	0.545	0.190	0.503	0.089	0.497	0.562	0.049
		(0.519 - 0.571)	(0.166 - 0.220)	(0.465 - 0.541)	(0.084 - 0.095)	(0.434 - 0.549)	(0.453 - 0.629)	(0.046 - 0.052)
**COST-SENSITIVE RF W/ SMOTE**	Test	0.530	0.392	0.650	0.087	0.351	0.726	0.047
		(0.509 - 0.564)	(0.255 - 0.486)	(0.455 - 0.895)	(0.079 - 0.101)	(0.156 - 0.689)	(0.429 - 0.929)	(0.041 - 0.053)
	Train	0.555	0.323	0.568	0.077	0.432	0.564	0.042
		(0.545 - 0.566)	(0.276 - 0.394)	(0.424 - 0.702)	(0.075 - 0.081)	(0.173 - 0.567)	(0.415 - 0.707)	(0.041 - 0.043)
**COST-SENSITIVE RF W/ ADASYN**	Test	0.511	0.509	0.915	0.080	0.085	0.976	0.042
		(0.502 - 0.527)	(0.486 - 0.521)	(0.891 - 0.935)	(0.078 - 0.083)	(0.051 - 0.104)	(0.929 - 1.000)	(0.041 - 0.043)
	Train	0.529	0.307	0.871	0.083	0.129	0.904	0.044
		(0.510 - 0.548)	(0.185 - 0.474)	(0.841 - 0.917)	(0.080 - 0.086)	(0.099 - 0.173)	(0.846 - 0.986)	(0.042 - 0.045)
**COST-SENSITIVE RF W/ SMOTEENN**	Test	0.510	0.175	0.874	0.078	0.126	0.905	0.041
		(0.501 - 0.518)	(0.140 - 0.221)	(0.801 - 0.915)	(0.074 - 0.081)	(0.075 - 0.199)	(0.786 - 1.000)	(0.039 - 0.042)
	Train	0.523	0.238	0.743	0.084	0.257	0.777	0.044
		(0.509 - 0.537)	(0.183 - 0.303)	(0.657 - 0.830)	(0.084 - 0.084)	(0.172 - 0.427)	(0.612 - 0.861)	(0.044 - 0.045)
**COST-SENSITIVE RF W/ RUS**	Test	0.503	0.086	0.894	0.078	0.106	0.928	0.041
		(0.501 - 0.505)	(0.057 - 0.115)	(0.816 - 0.948)	(0.076 - 0.079)	(0.062 - 0.184)	(0.821 - 1.000)	(0.040 - 0.041)
	Train	0.513	0.135	0.817	0.085	0.183	0.874	0.045
		(0.503 - 0.522)	(0.080 - 0.190)	(0.712 - 0.891)	(0.085 - 0.086)	(0.067 - 0.257)	(0.746 - 0.959)	(0.044 - 0.045)

RF: Random Forest, SMOTE: Synthetic Minority Over-sampling Technique, ADASYN: Adaptive Synthetic, RUS: Random under-Sampling, SMOTEENN: Synthetic Minority Over-sampling Technique Edited Nearest Neighbor, AUC-ROC: Area Under the Curve – Receiver Operative Curve, AUC-PR: Area Under the Curve – Precision-Recall Curve, w/: with, w/o: without, prob.cal.: probability calibration. Results obtained on test and training sets. For each metric mean value and its 95% confidence interval are reported.

TABLE S8 ENHANCED ADAPTIVE BOOSTING WITH DATA AUGMENTATION PERFORMANCE

MODEL		AUC-ROC	AUC-PR	BRIER SCORE	F1 SCORE	ACCURACY	RECALL	PRECISION
**ADABOOST W/ SMOTE W/O PROB. CAL.**	Test	0.521 (0.514 - 0.528)	0.141 (0.046 - 0.316)	0.531 (0.411 - 0.755)	0.087 (0.075 - 0.100)	0.469 (0.245 - 0.607)	0.619 (0.429 - 0.893)	0.047 (0.040 - 0.052)
	Train	0.550 (0.524 - 0.576)	0.109 (0.046 - 0.203)	0.331 (0.312 - 0.345)	0.108 (0.078 - 0.149)	0.669 (0.647 - 0.683)	0.437 (0.244 - 0.557)	0.062 (0.045 - 0.086)
**ADABOOST W/ ADASYN W/O PROB. CAL.**	Test	0.519 (0.513 - 0.525)	0.189 (0.097 - 0.252)	0.710 (0.612 - 0.858)	0.077 (0.072 - 0.082)	0.290 (0.142 - 0.437)	0.726 (0.536 - 0.929)	0.041 (0.039 - 0.043)
	Train	0.557 (0.525 - 0.589)	0.260 (0.258 - 0.264)	0.426 (0.407 - 0.444)	0.098 (0.080 - 0.121)	0.574 (0.542 - 0.598)	0.515 (0.367 - 0.599)	0.054 (0.044 - 0.067)
**ADABOOST W/ SMOTEENN W/O PROB. CAL.**	Test	0.512 (0.503 - 0.521)	0.117 (0.018 - 0.216)	0.405 (0.095 - 0.576)	0.051 (0.000 - 0.079)	0.595 (0.435 - 0.905)	0.381 (0.000 - 0.571)	0.028 (0.006 - 0.042)
	Train	0.530 (0.511 - 0.549)	0.187 (0.152 - 0.231)	0.379 (0.333 - 0.417)	0.087 (0.064 - 0.110)	0.621 (0.554 - 0.659)	0.421 (0.178 - 0.569)	0.049 (0.036 - 0.062)
**ADABOOST W/ RUS W/O PROB. CAL.**	Test	0.507 (0.504 - 0.511)	0.299 (0.260 - 0.366)	0.520 (0.367 - 0.618)	0.079 (0.072 - 0.087)	0.480 (0.339 - 0.633)	0.536 (0.429 - 0.679)	0.043 (0.038 - 0.048)
	Train	0.536 (0.516 - 0.556)	0.198 (0.168 - 0.229)	0.503 (0.393 - 0.572)	0.092 (0.086 - 0.098)	0.497 (0.398 - 0.567)	0.564 (0.486 - 0.634)	0.050 (0.047 - 0.055)
**ADABOOST W/ SMOTE W/ PROB. CAL.**	Test	**0.529 (0.505 - 0.554)**	0.172 (0.074 - 0.255)	0.422 (0.294 - 0.600)	0.082 (0.059 - 0.097)	0.578 (0.400 - 0.680)	0.476 (0.286 - 0.786)	0.046 (0.039 - 0.052)
	Train	0.550 (0.530 - 0.570)	0.109 (0.062 - 0.180)	0.367 (0.312 - 0.406)	0.095 (0.074 - 0.117)	0.633 (0.573 - 0.671)	0.438 (0.238 - 0.564)	0.054 (0.041 - 0.067)
**ADABOOST W/ ADASYN W/ PROB. CAL.**	Test	0.510 (0.503 - 0.517)	0.117 (0.015 - 0.196)	0.526 (0.317 - 0.741)	0.081 (0.073 - 0.087)	0.474 (0.259 - 0.615)	0.571 (0.357 - 0.857)	0.044 (0.041 - 0.048)
	Train	0.520 (0.509 - 0.531)	0.151 (0.142 - 0.162)	0.415 (0.394 - 0.435)	0.089 (0.075 - 0.103)	0.585 (0.547 - 0.605)	0.462 (0.356 - 0.516)	0.049 (0.041 - 0.057)
**ADABOOST W/ SMOTEENN W/ PROB. CAL.**	Test	0.518 (0.502 - 0.544)	0.256 (0.146 - 0.342)	0.604 (0.441 - 0.837)	0.080 (0.074 - 0.086)	0.396 (0.163 - 0.528)	0.655 (0.429 - 0.964)	0.043 (0.040 - 0.045)
	Train	0.557 (0.530 - 0.584)	0.289 (0.248 - 0.345)	0.479 (0.416 - 0.514)	0.091 (0.075 - 0.110)	0.521 (0.480 - 0.555)	0.542 (0.400 - 0.645)	0.050 (0.041 - 0.060)
**ADABOOST W/ RUS W/ PROB. CAL.**	Test	0.512 (0.511 - 0.512)	0.135 (0.065 - 0.272)	0.468 (0.214 - 0.651)	0.103 (0.083 - 0.141)	0.532 (0.235 - 0.786)	0.595 (0.429 - 0.893)	0.059 (0.045 - 0.079)
	Train	0.553 (0.539 - 0.567)	0.163 (0.146 - 0.188)	0.393 (0.360 - 0.426)	0.076 (0.054 - 0.090)	0.607 (0.564 - 0.663)	0.412 (0.327 - 0.500)	0.043 (0.030 - 0.052)

AdaBoost: Adaptive Boosting, SMOTE: Synthetic Minority Over-sampling Technique, ADASYN: Adaptive Synthetic, RUS: Random under-Sampling, SMOTEENN: Synthetic Minority Over-sampling Technique Edited Nearest Neighbor, AUC-ROC: Area Under the Curve – Receiver Operative Curve, AUC-PR: Area Under the Curve – Precision-Recall Curve, w/: with, w/o: without, prob.cal.: probability calibration. Results obtained on test and training sets. For each metric mean value and its 95% confidence interval are reported.
